# An integrative multi-omics investigation into the influence of forage type on the volatile flavor profile of Ujumqin sheep mutton

**DOI:** 10.3389/fvets.2026.1856240

**Published:** 2026-06-16

**Authors:** Wu Sachula, Lv Huimin, Zhao Yaxing, Yang Ding, Zhang Shangxiong, Li Shengli, Sun Haizhou, Zhang Chunhua

**Affiliations:** 1 Inner Mongolia Academy of Agriculture and Animal Husbandry Sciences, Hohhot, China; 2 Key Laboratory of Grass-Feeding Livestock Healthy Breeding and Livestock Product Quality Control (Co-Construction by Ministry and Province), Ministry of Agriculture and Rural Affairs, Hohhot, China; 3 Inner Mongolia Key Laboratory of Herbivore Nutrition Science, Hohhot, China; 4 National Technology Innovation Center for Prataculture, Hohhot, China

**Keywords:** alfalfa hay, corn stalk, flavor-omics, *Leymus chinensis* hay, metabolomics, oat hay, rumen microorganism

## Abstract

China ranks among the leading producers and consumers of mutton globally and the development of nutritional strategies to improve meat quality and sensory attributes. This study investigated the effect of three high-quality forages, i.e., alfalfa hay (ALFA), *Leymus chinensis* hay (LEYM) and oat hay (OATS) compared to corn stalks-based control diet (CORN) on rumen microbiota, metabolomics profiles, and muscle volatile flavor compounds in lambs through a multi-omics integration approach. Forty male lambs were randomly allocated into four dietary groups (n = 10/group) and fed a concentrated forage supplement for 91 days. From each group, six lambs (n = 6/group; totla 24) were slaughtered. Rumen fluid and longissimus dorsi muscle samples were collected for metagenomics, untargeted metabolomics, and volatile flavor analysis. Differential microbial taxa were identified using LEfSe analysis, followed by integrated Pearson correlation and MetoOrigin analysis to link microbiota, metabolites, and metabolic pathways. Associations with muscle volatile flavor compounds were also assessed. LEfSe analysis identified 4, 3, and 7 differentially abundant rumen microbial taxa in the ALFA, LEYM and OATS groups, respectively, compared to CORN. Integrated analysis showed these taxa correlated with 4, 9 and 11 rumen metabolites via 3, 11 and 7 microbial or host-microbial co-metabolic routes, respectively. These metabolic changes were strongly associated to alterations in muscle volatile flavor compounds. Particularly, the ALFA diet increased volatile compounds associated with fresh, grassy, floral, and citrus-like odors reduced mutton-related Pyrazine (2,5-dimethyl-). The LEYM diet reduced Pentaborane(9) and Pyrazine, which are associated with undesirable mutton like odors. The OATS diet increased 2-Nonanone and Phenylethyl Alcohol (fruity and floral smells), while suppressing n-Decanoic acid and n-Octanoic acid (associated with characteristic mutton aroma). These results showed that high-quality forages improve the mutton flavor by regulating the rumen micro-ecological network and associated metabolic pathways along the forage-microbiota-metabolites-muscle flavor axis. These findings provide a theoretical foundation for precise nutritional interventions aimed at enhancing meat quality in lambs.

## Graphical abstract

**Figure:**
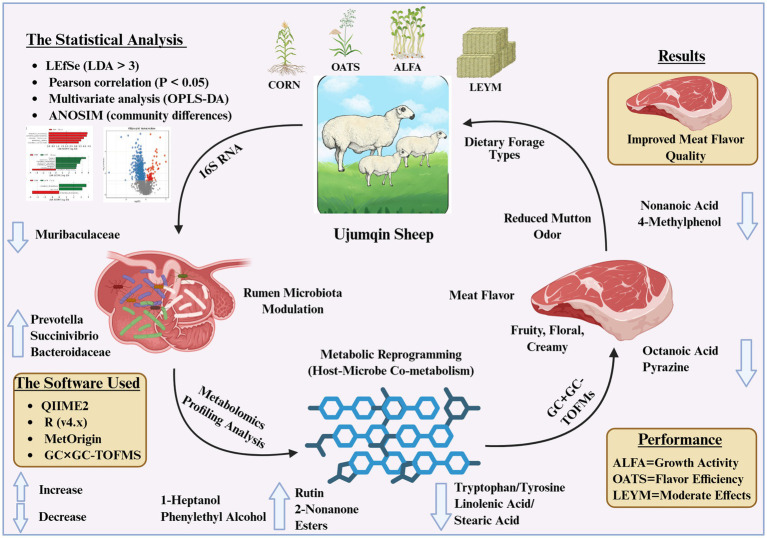

## Introduction

Lamb, characterized by its high protein content and low cholesterol levels, has become an important component of global meat consumption (1). Diet modification of livestock, has emerged as an effective strategy to improve growth performance, carcass traits and meat flavor, thereby addressing the rising demand for high-quality meat (2, 3). In contemporary ruminant production systems, producers employ a feeding strategy that integrates natural forage with concentrated feed to optimize growth performance, carcass traits, and meat quality. Previous studies demonstrate that natural forage diets can improve the meat quality while integrated diets of natural forage and concentrated feed can enhance production performance of lambs (4). The diet composition and quality are the critical dynamics of carcass composition, rumen microbiota, and the nutritional value of meat (5, 6).

Corn stalks, are widely available and low cost forage source of roughage in China and are used in ruminant feeding. However, they are classified as low-quality roughage, due to their high neutral detergent fiber (NDF) content (70–80%), a crude protein content (3–6%), low metabolic energy and poor digestibility (7). Previous work demonstrated that diet with high proportion of corn stalks can affect rumen microbial composition. For example 30% corn stalk diets in dairy cow reduce the relative abundance of *Treponema* species including *Treponema saccharophilum* and *Treponema succinifaciens,* resulting in downregulation of gene expression encoding succinate dehydrogenase ultimately reducing the deposition of flavor metabolites in meat (8). *Leymus chinensis (Trin. ex Bunge) Tzvelev*, a perennial herb belonging to the *Poaceae* family and the genus *Leymus*, is widely distributed across the eastern region of the Inner Mongolia Plateau, the Songnen Plain, and various other Eurasian steppe areas (9). This grass species has long been recognized as a valuable forage resource due to its high yield, suitable nutritional value and favorable palatability (10).

Research indicates that partial replacement of corn silage and alfalfa hay with sheep grass hay may enhance milk production, as well as the protein and milk fat concentration (11). Moreover, the sheep grass hay diet elevates the C15:0 fatty acids level in lamb meat as compared to a mixed feed diet (6). *Medicago sativa* is recognized comprehensive nutritional profile, excellent palatability, balanced amino acid composition and richness in vitamins and minerals. With abundancy in bioactive compounds, the roots, stems, and leaves of this forage, globally known as the “King of Forage,” exhibit antioxidative properties and enhance immune function (12, 13). The inclusion of alfalfa hay significantly enhances the growth performance and milk production of lambs as compared to wheat straw diet (14). Moreover, supplementation with high-quality alfalfa hay can purposefully can the omega-3 fatty acid content in mutton and reduce the omega-6/omega-3 ratio while improving its nutritional value (15). Oat (*Avena sativa* L.), another widely used high quality feed diet, are recognized globally by its high protein content and feed digestibility and strong adaptability and nutritional value (16). Previous studies indicate the supplementary consumption of oat grass can positively influences feed efficiency and livestock performance, resulting in improved food intake, body weight, rumen fermentation and meat quality (17, 18). These diets demonstrated a significant increase in the relative abundance of short-chain fatty acid (SCFA) producing bacteria, including *Bacteroidetes*, *Fibrobacteres*, and *Prevotellaceae*, in the rumen of oat hay fed sheep (19). Additionally, studies have also shown that Oat grass supplementation can increase the body and carcass weight in sheep and amino acid content in muscle, thereby improving the quality and flavor of mutton (20).

Muscle quality is closely connected to the metabolism and digestion of dietary components within the rumen. The rumen contains multifaceted and dynamic microbial ecosystem comprising the host bacteria, eukaryotic microorganisms, archaea, and other microbial entities. These microbes support the feed fermentation, convert fiber-rich plant materials (21), form a mutualistic relationship with the host and supply ruminants’ energy requirements (22, 23). Some strong evidences also emphasize the interaction between microorganisms and muscle metabolites. For example, the relative abundance of microbiota, such as *Prevotella* UCG-003 in the rumen, shows a significant positive correlation with key fatty acids, including linoleic acid (C18:2n6c), gamma-linolenic acid (C20:3n6), and palmitic acid (C16:0) in muscle tissue (24). Moreover, amino acids like isoleucine and glycine in the rumen are positively associated with *Anaeroplasma*, while showing a negative association with *Parabacteroides* and *Alloprevotella* (25). The rumen microbiota plays a vital role in the rumen and communicates with the host through various metabolites (26). The studies also demonstrated the positive relationship among the rumen microbiota, fatty acid metabolites and flavor compounds between EPA and DHA levels in the rumen and the amounts of EPA and n-3 PUFAs in muscle tissue (27). This study suggests that the rumen functions not only as an organ for digestion and fermentation of the diet but also as a site for synthesizing precursors of flavor. Therefore, elucidating the interaction between the rumen microbiome and muscle volatile metabolites may provide new insights into the microbiota-driven flavor metabolism (28).

The dietary roughage types play roles in influencing the content and composition of muscle metabolites in lamb, and affect the meat flavor. Previous studies explained the substitution of cottonseed hulls with woody plants, such as red berry juniper, in the lambs’ diets. This dietary change increases an oleic acid content in the intermuscular fat of lambs by 4.2 to 8.0%, while the stearic acid content decreased by 0.3 to 2.8%, which slightly enhanced the meat flavor (29). Similarly, the consumption of different roughages by lambs alters the muscle fatty acid content like alfalfa hay increases the C14:0 fatty acid content, whereas wheat stubble improves the C18:0 content in the lambs’ muscles (30). The feeding of low-energy cabbage and alfalfa can improve the lamb flavor (31). Previous studies have primarily focused the effects of individual forage grasses on the conventional quality of livestock meat, without thoroughly exploring their metabolic flavor profiles. To understand the underlying mechanisms, it is essential to integrate multiple omics techniques to clarify the relationships among forage, rumen, and muscle. Previous researches have been focused primarily on growth performance, carcass traits, fatty acid composition, or general meat quality traits, and the interaction mechanism between forage type, rumen microbiota, rumen metabolites and muscle volatile flavor compounds remains poorly understood (32). Specifically, few studies have combined metagenomics, metabolomics, and flavor-omics to reveal the microbial and metabolic mechanism of mutton flavor regulation (33). Based on this, the present study integrated multi-omics approach to systematically characterize forage–rumen microbiota – metabolite – muscle flavor axis and identify key microbial taxa, metabolic pathways, flavor-associated metabolites associated with improved sensory quality of mutton.

This study aims to determine the relationship between high-quality forage and the flavor metabolites in the sheep muscle, mediated by rumen microorganisms. This investigation assesses the effects of supplementing high-quality forage, such as alfalfa, oat and sheep grass, on volatile flavor compounds, rumen microbial communities and metabolites in the longissimus dorsi muscle of sheep, comparing the supplementation of low-quality roughage, specifically corn stalks. To achieve this, an integrated multi-omic approach was employed combining metagenomic sequencing with non-targeted metabolomics to identify different microorganisms and metabolites in rumen fluid, including precursors associated with mutton-like odor. Flavor omics techniques were employed to analyze volatile flavor compounds in the longissimus dorsi muscle, allowing for the screening of different flavor metabolites and the identification of characteristic flavor substances. Collectively, the study analyzed the correlations between volatile metabolites related to muscle and mutton-like flavor characteristics, alongwith the differential microorganisms and metabolites present in the rumen. This analysis provides a theoretical foundation for the judicious application of high-quality forage aimed at improving meat quality in lamb production system.

## Materials and methods

### Experimental design and sample collection

A total of 40 healthy weaned male meat sheep lambs of 60 days’ age and 25.66 ± 3.03 kg weight, were used in this study. The experiment followed a single-factor completely randomized design (CRD) in which lambs were randomly assigned to four distinct forage treatment groups. All lambs received a uniform amount of concentrated feed to meet energy levels while having unlimited access to one type of forage: corn stalks (CORN), alfalfa hay (ALFA), oat hay (OATS) or *Leymus chinensis* hay (LEYM). The experiment included a one-week pre-feeding period and a 91-day formal period. Each lamb was fed twice a day in the morning and afternoon, and fed 0.5 kg/day of concentrate equally to each lamb. Water and the specified forage were ad libitum and the concentrate diet was formulated as shown in Table 1.

**Table 1:** Ingredient composition of the concentrate diet used in the experimental treatments (%, as-fed basis).

Item	Usage amount (%)
Raw material	
Corn kernels	52.6%
Soybean meal	18.6%
Sesame meal	5.5%
Fine bran	10.8%
Rice bran meal	7.5%
Stone powder	1.6%
CaHPO_4_	0.65%
NaCl	0.5%
Premix	2.25%
Total	100%
Nutrient content	
ME MJ/kg	10.664
DM %	82.7
CP %	17.96
NDF %	23.67
ADF %	15.45
Ca %	1%
P %	0.6%

This formulation was designed to meet the nutrient requirements of growing lambs. The premix supplied vitamins and trace minerals (Each kg of premix containing the following: 560000 IU of Vitamin A, 60000 IU of Vitamin D, 600 IU of Vitamin E, 600 mg of Cu, 3800 mg of Zn, 2600 mg of Fe, 3800 mg of Mn, 24 mg of Se, 56 mg of I and 56 mg of Co). CaHPO₄ represents calcium hydrogen phosphate.

The entire experimental procedure was approved by the Research Ethics Committee of the Inner Mongolia Autonomous Region Academy of Agricultural and Animal Husbandry Sciences (Approval No. 2024–0020) and were conducted according to established animal welfare protocols. Following the experiment, 24 sheep were randomly selected (n = 6 per treatment), fasted for 12 h, and then slaughtered. After slaughtering, the longissimus dorsi muscle and rumen fluid were collected immediately. Muscle samples were excised from the same location in the longissimus dorsi muscle, between the 10th and 13th ribs on the left carcass of each lamb, using a sterile disposable scalpel. Until further analysis, the samples were preserved in liquid nitrogen at −80 °C. In rumen sampling, the rumen contents were sampled by removing them after opening the rumen and passing them through a four-layers of sterile gauze. Approximately, 50 mL of rumen fluid was transferred into 50 mL RNase-free centrifuge tubes having RNase inhibitors and stored at −80 °C for subsequent multi-omics analysis.

### Growth performance and muscle quality assessment

During the experimental period, feed intake, daily weight gain, and feed conversion ratio (F/G) of sheep were recorded for each treatment group. Following slaughter, meat quality traits of LD muscle were evaluated. Muscle colour was measured using a colorimeter (HunterLab MiniScan EZ, USA). Drip loss was calculated using standardized muscle sample (1 cm × 1 cm × 2 cm) suspended at 4 °C for 24 h and calculated as percentage weight loss before and after storage. A 100 g sample of LD was steamed for 30 min at a core temperature of 70 °C and subsequently cooled to room temperature for cooking loss determination. Cooking drip loss was calculated based on difference in sample weight before and after steaming. The samples were then further cooled to 4 °C prior to texture analysis. Shear force was evaluated to measure meat tenderness. Three cylindrical cores were removed parallel to longitudinal muscle direction and shear force was determined perpendicular to fiber orientation using a texture analyzer (C-LM4, Tenovo International Co., Limited).

### Sample determination and data statistical analysis

Metagenomic sequencing of rumen fluid, rumen metabolites assessment, and volatile flavor analysis of the longissimus dorsi muscle were performed by Suzhou Panomic Biomedical Technology Co., LTD. The rumen fluid metagenome was sequenced using the llumina NovaSeq platform, the rumen metabolites were detected and confirmed by LC–MS / MS, and the volatile flavor compounds of longissimus dorsi muscle were detected and confirmed by GCxGC TOF MS. Data processing and bioinformatics analysis were conducted using the BioDeep cloud platform.^1^ All data were analyzed employing one-way analysis of variance (ANOVA) via SPSS 24 (IBM, USA) software. Differences among the data were assessed using ANOVA followed by multiple comparison test. Pearson correlation analysis was conducted to determine possible relationships among the microbial taxa, rumen metabolites and volatile flavor compounds. Correlations with |r| > 0.5 and *p* < 0.05 were considered biologically meaningful moderate-to-strong associations, consistent with previously published exploratory multi-omics integration studies (34). Statistical analysis and data visualization were performed using R software.

## Results

### Effects on growth performance and muscle quality

The growth and meat quality traits of 24 lambs were measured. There were significant differences in average daily feed intake, average daily weight gain, meat color (b*), water loss and cooking loss among different forage groups (*p* < 0.05) as shown in Table 2. In terms of growth performance, the average daily feed intake and average daily total weight gain of the high-quality forage group (LEYM, OATS, ALFA) were significantly higher than those of the CORN group, and the feed-to-weight ratio of the ALFA group was lower than that of other groups. In terms of meat quality, feeding the OATS group significantly reduced cooking loss, feeding LEYM group significantly reduced drip loss and muscle yellowness (b*) at 30 min of slaughter (*p* < 0.05).

**Table 2:** Effects of dietary forage type on growth performance and physicochemical properties of lamb meat.

Items (mean ± SD)	Group				*p*-value
	CORN	LEYM	OATS	ALFA	
Average daily feed intake (kg/day)	1.0994 ± 0.044^c^	1.149 ± 0.018^b^	1.1702 ± 0.041^b^	1.4487 ± 0.024^a^	0
Average daily gain (g/d)	66.942 ± 27^b^	89.863 ± 49^b^	80.928 ± 36^b^	164.43 ± 33^a^	0.001
Feed-to-weight ratio F/G	18.58 ± 6.67	18.42 ± 12.87	16.74 ± 7.05	9.09 ± 1.73	0.173
cooking loss, %	39.36 ± 1.32^a^	34.47 ± 4.18^a^	21.84 ± 12.91^b^	34.48 ± 1.94^a^	0.002
Shear force (N)	48.05 ± 14.48	54.99 ± 12.55	58.06 ± 11.61	58.58 ± 10.91	0.455
Drip loss, %	1.13 ± 0.29^bc^	0.98 ± 0.36^c^	1.65 ± 0.82^a^	1.51 ± 0.29^ab^	0.005
L* 30 min	34.04 ± 4.09	31.32 ± 2.89	33.57 ± 1.75	33.17 ± 3.93	0.21
a* 30 min	12.93 ± 1.34	13.20 ± 1.27	13.84 ± 0.79	13.62 ± 1.42	0.275
b* 30 min	9.23 ± 1.39^a^	8.14 ± 1.07^b^	9.93 ± 0.78^a^	10.11 ± 1.35^a^	0.001

Values are stated as mean ± SD. Means within a row with different superscript letters (a–c) differ significantly (*p* < 0.05). CORN represents the control diet based on corn stalks while LEYM, OATS, and ALFA indicate diets supplemented with *Leymus chinensis*, oat hay, and alfalfa hay, respectively. Growth performance indicators include ADFI (kg/day), ADG (g/day), and feed conversion ratio (F/G). Meat quality traits include cooking loss (%), shear force (N), drip loss (%), and color parameters (L*, lightness; a*, redness; b*, yellowness) determined at 30 min postmortem.

### The diversity and composition of rumen microorganisms

#### Sample diversity

**Figure 1:**
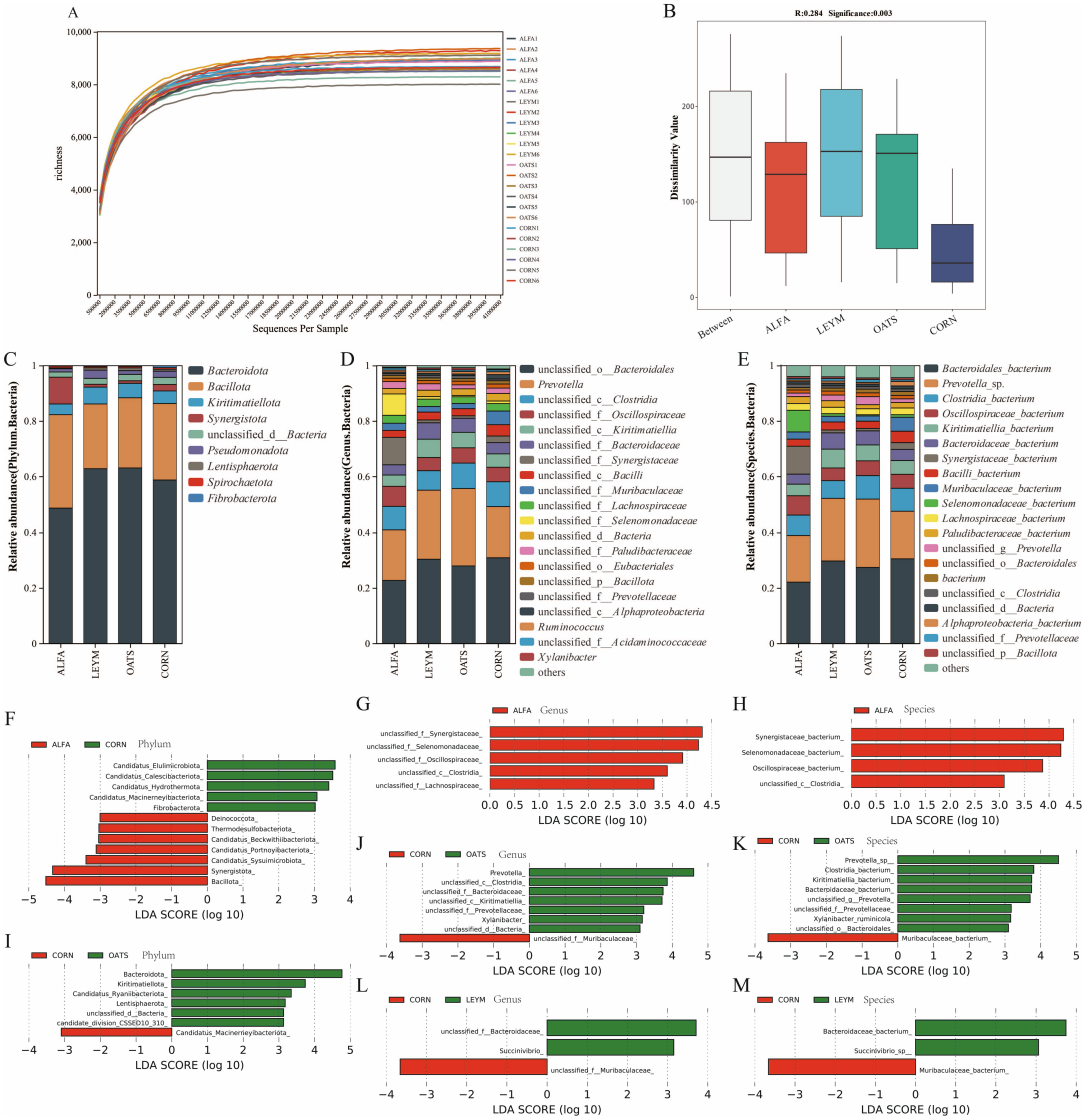
Diversity and taxonomic composition of rumen bacterial communities under different dietary treatments. **(A)** Rarefaction curves illustrating sequencing depth and species richness across samples, indicating sufficient coverage of microbial diversity. **(B)** Analysis of similarities (ANOSIM) showing differences in bacterial community structure among dietary groups. **(C–E)** Relative abundance of rumen bacteria at different taxonomic levels: **(C)** phylum level, **(D)** genus level, and **(E)** species level. **(F–M)** LEfSe analysis identifying significantly enriched bacterial taxa among groups (LDA > 3), highlighting diet-associated microbial biomarkers.

Rumen fluid samples from 24 lambs were sequenced metagenomically using the Illumina NovaSeq 6,000 platform. A total of 1,284,489,118 original sequences (raw reads) were generated from 24 rumen fluid samples, yielding an average of approximately 53.52 million reads per sample. High-quality clean reads were obtained and then assembled after quality control featuring read filtering and trimming. Assembly and scaffolding yielded 10,958,959 contigs, with an average of 438,358.36 contigs per sample. After gene prediction and de-redundancy, total non-redundant genes identified were 3,147,644, with an average of 125,905.76 genes per sample. Taxonomic annotation based on the NR database identified 21,850 annotated microbial taxa within the rumen metagenomics dataset. The microbial community was directed by bacteria (73.7%), eukaryotic microorganisms (21.2%), archaea (2.1%) and other organisms (2.8%). The dilution curves for all sample reached a plateau, suggesting sufficient sequencing (Figure 1A). In terms of *α*-diversity indicators of the rumen microbiota (Supplementary Table S1), analysis based on taxonomic profiling and species abundance revealed no significant differences in the Simpson and Chao 1 indices between the two groups (*p* > 0.05). However, similarity analysis (ANOSIM) revealed that the gut microbiota composition varied among the four different forage groups (Figure 1B).

#### The impact on rumen bacteria

After analyzing the abundance of rumen bacteria, according to the species annotation, a total of 140 phyla, 2,883 genera and 9,385 species were identified. Among them, 8, 4 and 13 definite phyla, genera and species, respectively, were considered to be bacterial species with relatively high abundance (at least one group had a relative abundance >0.1%). At phylum level, the rumen microbiota was composed of *Bacteroidota* with relative abundance in ALFA (48.70%) vs. LEYM (62.89%) vs. OATS (63.14%) vs. CORN (58.81%). Secondly, there are *Bacillota* (33.61% vs. 23.27% vs. 25.23% vs. 27.50%) and *Kiritimatiellota* (3.84% vs. 6.06% vs. 5.20% vs. 4.54%) and Synergistota (9.63% vs. 1.03% vs. 0.91% vs. 2.30%) (Figure 1C). At the genus level, the most abundant was *Prevotella* across all groups accounting for 18.19, 24.84, 27.82 and 18.39% in ALFA, LEYM, OATS and CORN, respectively (Figure 1D). At species level, the most abundant ones were *Bacteroidales bacterium* (22.07, 29.68, 27.37 and 30.47%), *Prevotella* sp. (16.76, 22.52, 24.58 and 17.10%), *Clostridia bacterium* (7.37, 6.36, 8.44 and 8.26%), *Oscillospiraceae bacterium* (6.94, 4.57, 5.32, 5.01%) and *Kiritimatiellia bacterium* (4.16, 6.75, 5.71 and 4.96%) in ALFA, LEYM, OATS and CORN, respectively (Figure 1E).

To explain the differences in bacterial communities between the CORN and the other forage feeding groups, Line Discriminant Analysis Effect Size (LEfSe) was performed to identify discriminative taxa (LDA > 3, *p* < 0.05). In the comparing the ALFA and CORN, the abundance of bacterial taxa such as *Oscillospiraceae bacterium*, *Selenomonadaceae bacterium*, *Synergistaceae bacterium*, *Deinococcota*, *Thermodesulfobacteriota*, *Synergistota*, and *Bacillota* was significantly greater in the ALFA group while *Fibrobacterota* was significantly enriched in the CORN group (Figures 1F–H). In comparing OATS and CORN groups, *Xylanibacter ruminicola*, *Bacteroidaceae bacterium*, *Kiritimatiellia bacterium*, *Clostridia bacterium*, and *Prevotella* sp. were significantly higher in the OATS group, while *Muribaculaceae bacterium* was much abundant in the CORN group (Figures 1I–K). In the LEYM vs. CORN groups comparison, *Succinivibrio* sp. and *Bacteroidaceae bacterium* was significantly greater in the LEYM group, whereas *Muribaculaceae bacterium* was significantly abundant in the CORN group (Figures 1L,M).

#### The impact on rumen eukaryotes

**Figure 2:**
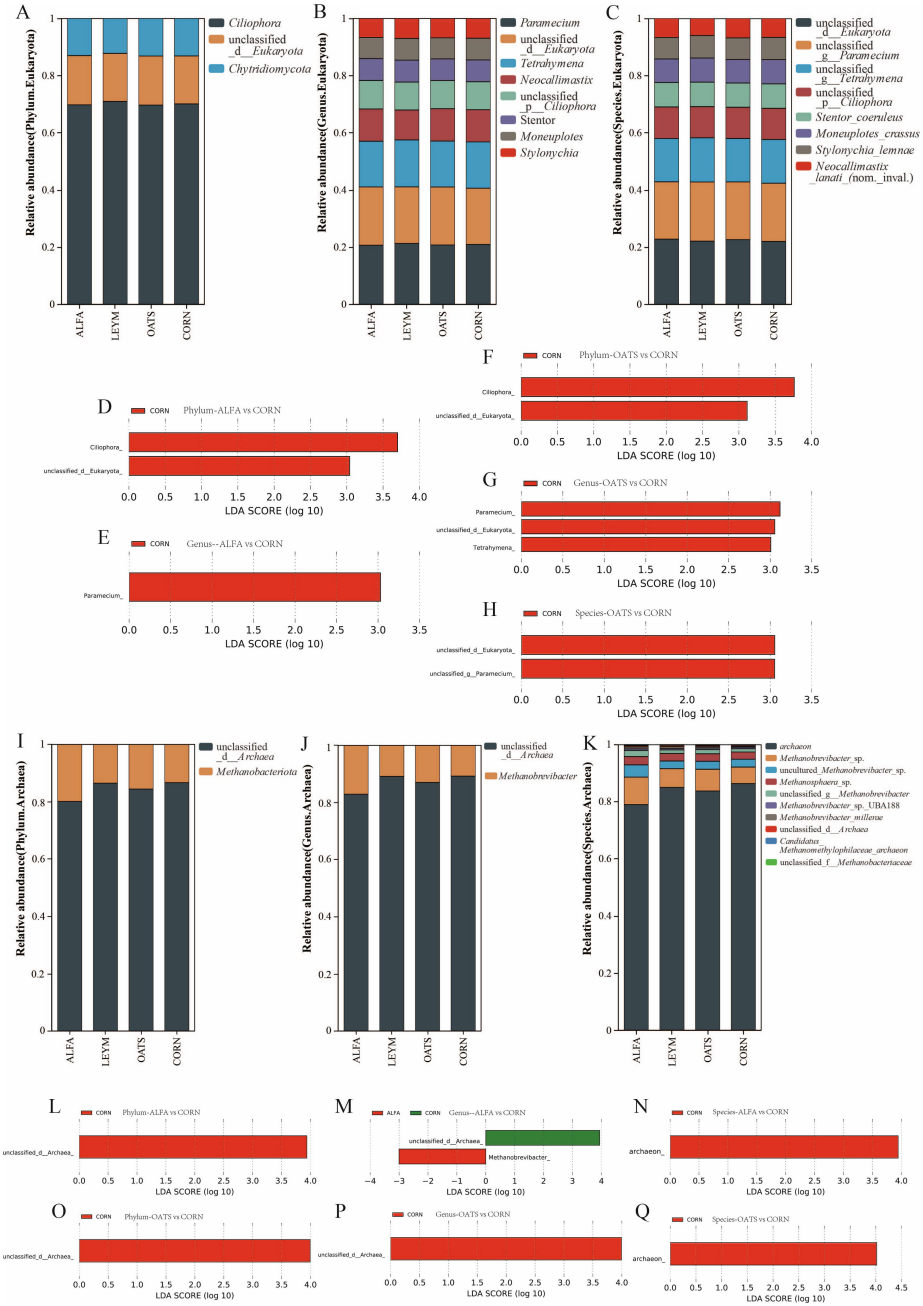
Composition and differential analysis of rumen eukaryotic microorganisms and archaea across dietary treatments. **(A–C)** Relative abundance of rumen eukaryotic microorganisms at **(A)** phylum, **(B)** genus, and **(C)** species levels. **(D–H)** LEfSe analysis of eukaryotic microorganisms shows taxa significantly enriched in each group (LDA > 3). **(I–K)** Relative abundance of rumen archaea at (I) phylum, **(J)** genus, and **(K)** species/strain levels. **(L–Q)** LEfSe analysis of archaeal communities identifies differentially abundant taxa among dietary treatments (LDA > 3).

A total of 26 phyla, 1,146 genera, and 2,171 species of rumen eukaryotic microorganisms were identified through species annotation analysis. Among the eukaryotic microbiota taxa with a relative abundance exceeding 0.1%, two dominant phyla were examined: *Ciliophora* with abundances of 69.73, 70.92, 69.64 and 70.06% in ALFA, LEYM, OATS and CORN and *Chytridiomycota* with relative abundances 13.05% vs. 12.27% (Figure 2A). Additionally, six dominant genera were identified, with a relative abundance of 13.18% vs. 13.16%. The most abundant genera included Paramecium at 20.64, 21.27, 20.75 and 20.92% and Tetrahymena at 15.99 and 16.43% across respective forage group (Figure 2B). Furthermore, four dominant species were annotated with relative abundances of 16.06% vs. 16.20% (Figures 2B,C).

LEfSe analysis was performed to compare the ALFA and CORN groups, within the *Ciliophora* phylum, the *Paramecium* was more abundant in the CORN group (LDA > 3, *p* < 0.05) (Figures 2D,E). Similarly, in the comparison of the OATS and CORN groups, both *Paramecium* and *Tetrahymena* in the *Ciliophora* phylum was significantly abundant in the CORN group than the OATS group (LDA > 3, *p* < 0.05) (Figures 2F–H). Conversely, in the LEYM and CORN groups analysis, no eukaryotic microorganisms were identified that met the criteria of LDA > 3 and *p* < 0.05.

#### The influence on the abundance of rumen archaea

The metagenomic sequences of archaea identified a total of 26 phylum, 179 genera, and 361 species with *Methanobrevibacter* exhibiting the highest abundance (Figures 2I–K). Analysis using LEfSe revealed that *Methanobrevibacter* was significantly more abundant in the ALFA group as compared to the CORN group (LDA > 3, *p* < 0.05) (Figures 2L–N). No archaea met the criteria of LDA > 3 and p < 0.05 in the OATS and CORN groups comparison (Figures 2O–Q). Furthermore, there were no significant differences in archaea with p < 0.05 observed in the LEYM versus CORN group.

### Rumen metabolomics research

The orthogonal partial least squares discriminant analysis (OPLS-DA) score plot exhibited a significant separation between the CORN and ALFA groups, as well as between the OATS and LEYM groups. Additionally, the OPLS-DA model validation plot explain that both the R^2^ and Q^2^ values are lower than their original values in the upper right corner. These observations suggest that the original model did not overfit and exhibited strong stability. Consequently, these findings confirm that the metabolic substances of lamb rumen had altered in response to the different forage types provided (Supplementary Figure 1).

**Figure 3:**
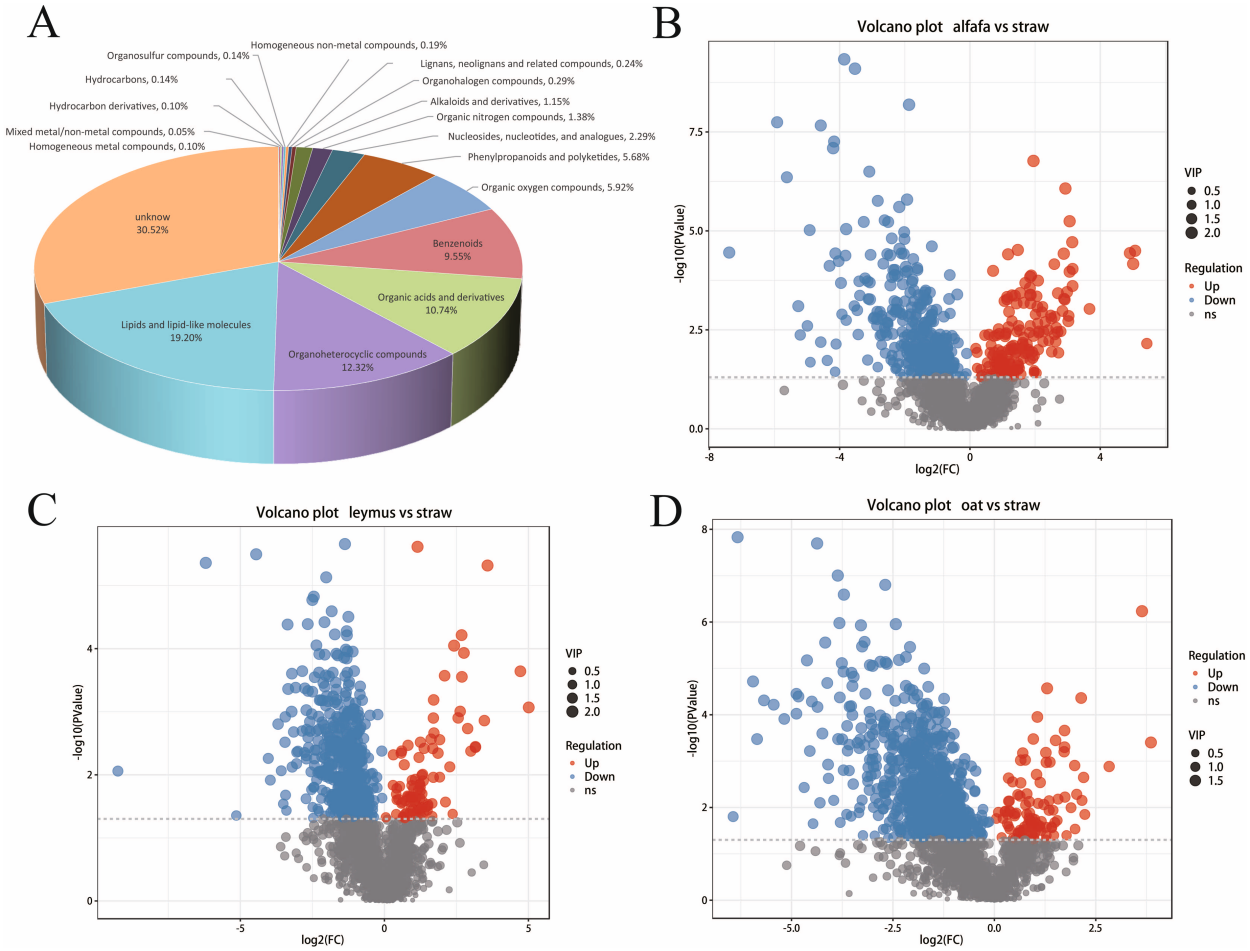
Rumen metabolomic profiling and identification of differential metabolites under different forage treatments. **(A)** Overview of rumen metabolite classification, showing distribution of annotated metabolites across major chemical categories. **(B–D)** Volcano plots illustrating significantly differential metabolites between groups (based on FC, VIP, and *p*-value thresholds), where red and blue dots represent upregulated and downregulated metabolites, respectively.

A total of 14,729 mass spectral characteristics of primary differential metabolites were extracted in both positive and negative ion modes through non-targeted metabolomics analysis of rumen metabolites. The metabolites identification was carried out by comparing MS/MS spectra with existing databases such as HMDB (35), LipidMaps (36) and KEGG (37), revealing 2094 annotated metabolites that were grouped into 18 chemical categories. The three categories with major proportions were Lipids and lipid-like molecules, organo-heterocyclic compounds, and organic acids and their derivatives (Figure 3A). Statistical analyses were conducted on the metabolites from each sample group to assess the effects of feeding four different forage grasses on rumen metabolites in sheep, employing both univariate and multivariate statistical methods (FC ≥ 2 or ≤0.5、*p* < 0.05 and VIP>1). Compared to the CORN group, the ALFA group, LEYM group, and OATS group exhibited 258 differential metabolites (174 down-regulated and 84 up-regulated), 239 differential metabolites (211 down-regulated and 28 up-regulated) and 426 differential metabolites (402 down-regulated and 24 up-regulated) respectively (Figures 3B–D).

### Analysis of flavor substances in the longissimus dorsi muscle

**Figure 4:**
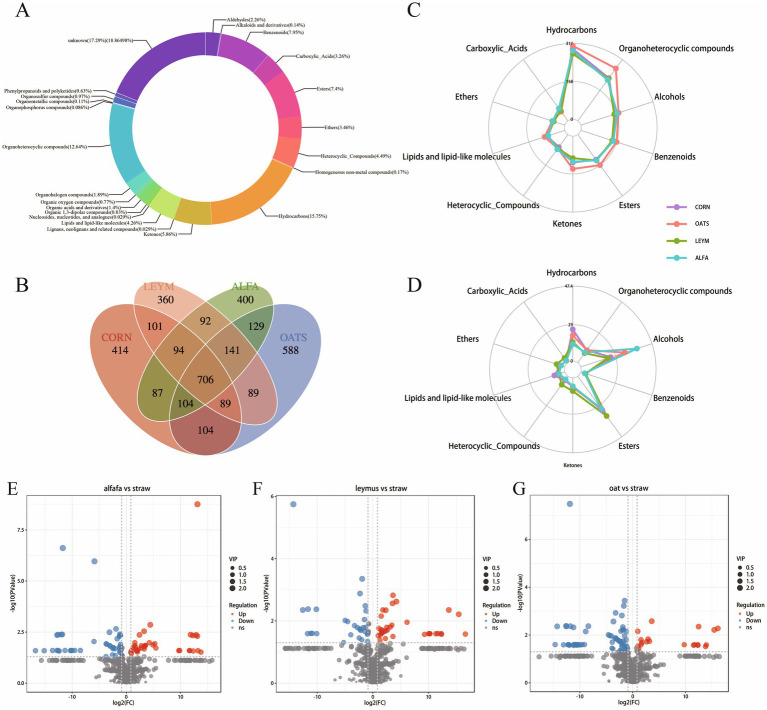
Profiling and comparative analysis of volatile flavor compounds in the longissimus dorsi muscle. **(A)** Classification and distribution of detected volatile flavor compounds across chemical categories. **(B)** Venn diagram showing shared and unique flavor compounds among different dietary groups. **(C)** Radar chart illustrating the number of different types of volatile compounds (Top 10 categories). **(D)** Radar chart representing relative abundance of volatile compound classes across groups. **(E–G)** Volcano plots showing differential volatile flavor compounds between treatment groups, indicating significantly upregulated and downregulated metabolites.

To understand the metabolite changes in meat flavor from sheep fed high-quality forage, GCxGC TOF MS full two-dimensional chromatography was employed to analyze the flavor compounds in the dorsal long muscle. The PubChem database and Classyfire software facilitated the type annotation analysis of the identified flavor substances. A total of 3,498 flavor substances were detected and categorized into 24 distinct groups. The five categories with the highest diversity included hydrocarbons (551; 15.75%), organic heterocyclic compounds (442; 12.64%), alcohols (291; 8.32%), benzene ring compounds (278; 7.95%), and esters (259; 7.40%) (Figure 4A).

The quantitative analysis across the groups exhibited that the identified volatile compounds in CORN, LEYM, ALFA and OATS groups were observed 1,699, 1,672, 1753 and 1950, respectively (Figure 4B) and specific flavor compounds were annotated 414, 360, 400 and 588, respectively. Among them, OATS group exhibited highest number of both flavor substances and specific flavor substances (Figure 4B). In number of substance types, OATS revealed greater number of 307 hydrocarbons, 271 organoheterocyclic compounds, 257 benzenoids, 158 esters, 137 ketones, and 89 lipids and lipid-like molecules (Figure 4C). Regarding number and content of substance types, the CORN group, the ALFA group and the LEYM group, respectively, possessed the richest contents of flavor metabolites related to hydrocarbons, alcohols and esters. The OATS group, however, showed a relatively rich content of flavor metabolites in hydrocarbons and alcohols (Figure 4D).

Multivariate statistical methods were employed to investigate the differences in flavor metabolites of sheep dorsal long muscle across various treatment groups. The CORN group served as the control for the statistical analysis and screening of metabolites between each pair of groups, as well as for the assessment of differential flavor metabolites among the control groups. Supplementary Figure 2 presents the OPLS-DA score plots, OPLS-DA permutation test plots and differential heat maps for comparisons between the CORN group and the ALFA, LEYM, and OATS groups, respectively. As illustrated in Supplementary Figures 2A–C, the control groups are distinctly separated from one another in pairs, and the Q^2^ values for all comparison groups exceed 0.6 (Supplementary Figures 2D–F), indicating that the constructed model is robust. The heat map (Supplementary Figures 2G–I) further describe that each group of samples can cluster independently, highlighting significant differences in the composition of flavor metabolites in the longus dorsal muscle of sheep fed different forages.

Following criteria for screening differential volatile compounds: FC > 2 or <0.5, *p* < 0.05 and VIP>1 were established to further investigate the alterations in volatile substances. As compared to the CORN group, the ALFA group identified a total of 77 differentially volatile compounds, containing 29 down-regulated and 48 up-regulated substances. The LEYM group identified 61 differential volatile compounds, with 34 down-regulated and 27 up-regulated while the OATS group exhibited 79 differential volatile compounds, consisting of 18 down-regulated and 61 up-regulated (Figures 4E–G).

**Figure 5:**
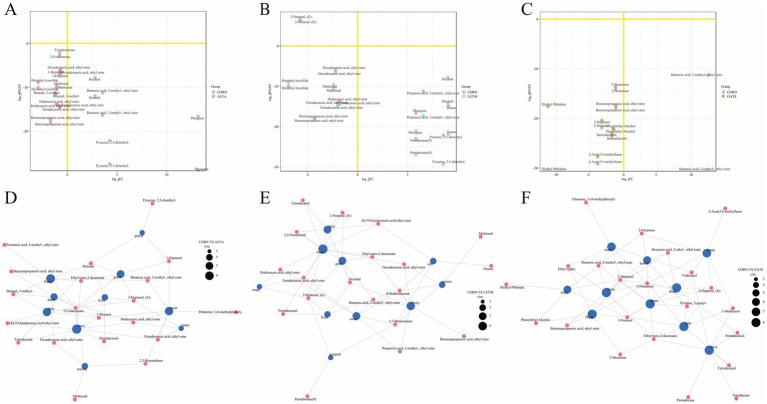
Identification of key flavor compounds and their association with sensory characteristics. **(A–C)** ROAV (Relative Odor Activity Value)–FC scatter plots showing the top 20 differential volatile compounds contributing to flavor differences among groups. **(D–F)** Correlation network analysis between sensory attributes and volatile compounds, where nodes represent compounds or sensory traits and edges indicate significant correlations.

In this study, the Flavordb database was utilized (cosylab.iiitd.edu.in/flavordb/) to analyze sensory flavor characteristics and identify differences among various groups. Relative odor activity (ROAV) analyzed 3,498 volatile flavor compounds at their respective volatile thresholds to identify flavors that influence sensory perception. This analysis revealed a total of 138 flavors that affect the senses, as detailed in Supplementary Table S2. Further investigation of the differential flavor metabolites indicated that the ALFA vs. CORN group, LEYM vs. CORN group, and OATS vs. CORN group were associated with 13 types of flavor substances (9 up-regulated and 4 down-regulated), 13 types (7 up-regulated and 6 down-regulated), and 8 types (7 upregulated) respectively. Figures 5A–C illustrates one category of differential flavor substances related to sensory perception.

Figures 5D–F demonstrates the association network diagram of sensory flavor characteristics and flavor substances across various control groups. In comparing the CORN with ALFA groups, the flavor metabolites influenced sensory perception including 1-Heptanol, having grassy aroma, and 2-Undecanone, characterized by a fresh orange scent; both were upregulated in the ALFA group. In the CORN versus LEYM group, Octane, associated with a gasoline flavor, was down-regulated in the LEYM group, whereas 2-Nonenal (E), known for its fatty and cucumber flavor, was upregulated in the LEYM group. Volatile flavor compounds were analyzed separately and exhibited specific changes in metabolites linked with sensory reactions in forage groups. Compounds linked with desirable sensory properties such as 2-nonanone (fruity/floral aroma), phenylethyl alcohol (floral aroma) and 2-heptanol (fruity aroma) were abundantly upregulated in the OATS group as compared to CORN. Conversely, those compounds were associated with the mutton-like odors, e.g., octanoic acid and n-decanoic acid, were suppressed in the OATS group. Benzenepropanoic acid ethyl ester was up-regulated across the ALFA, LEYM and OATS groups in comparison to CORN group. Moreover, various flavor-enhancing chemicals such as dimethyl trisulfide, hexadecanoic acid ethyl ester, tetradecanoic acid ethyl ester and dodecanoic acid ethyl ester were also highly expressed in the ALFA and LEYM groups. Conversely, the pyrazine, 2,5-dimethyl- associated with mutton-like odor, was suppressed in these groups.

### Correlation analysis

**Figure 6:**
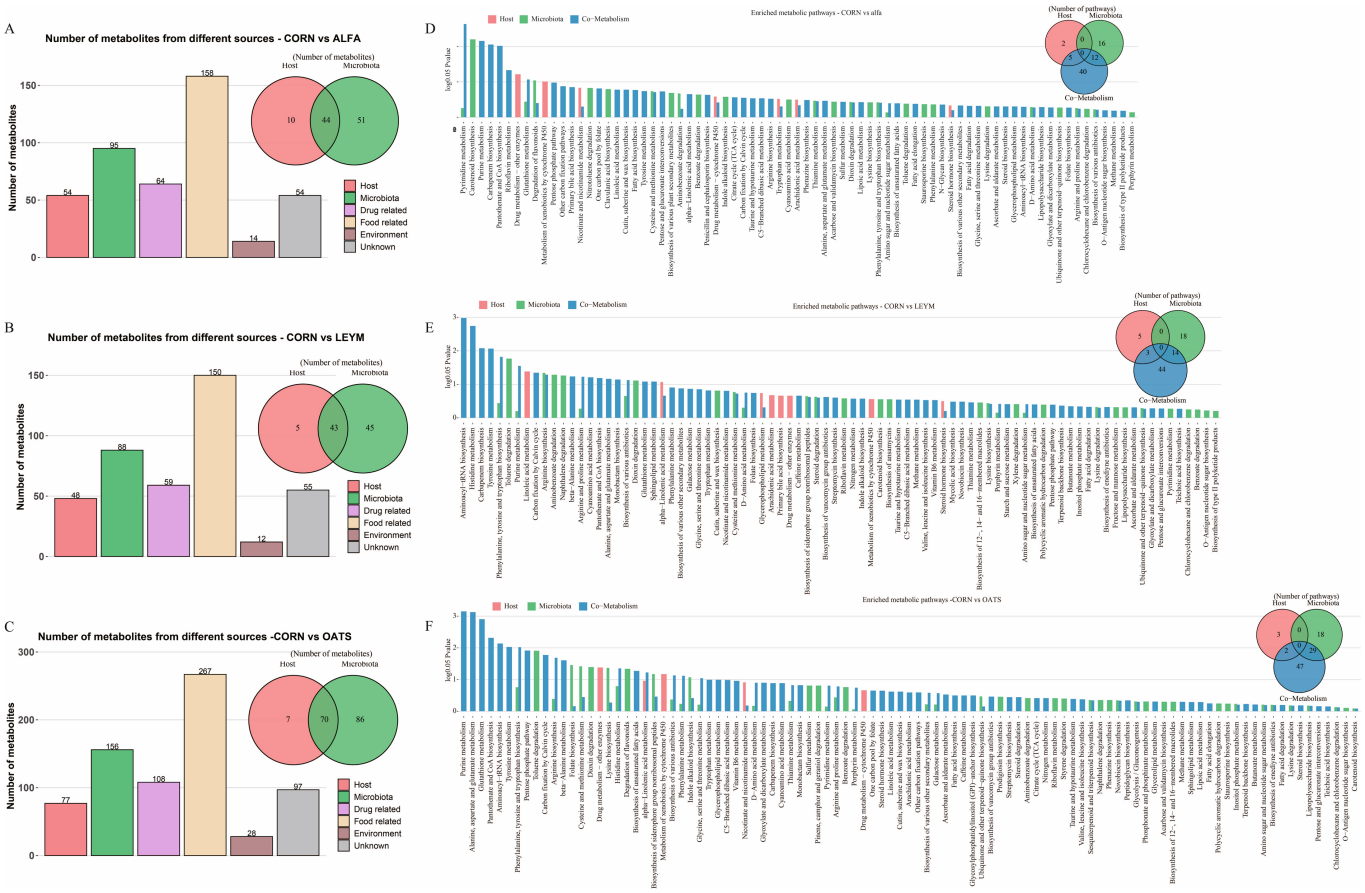
Source tracking and pathway enrichment analysis of differential metabolites. **(A–C)** Source analysis of differential metabolites, illustrating contributions from host metabolism, microbial metabolism, and co-metabolism. **(D–F)** KEGG pathway enrichment analysis showing significantly altered metabolic pathways associated with different dietary treatments.

The visual interactive online software MetOrigin were utilized to identify differential metabolites associated with microorganisms for our analysis. The metabolites origins were initially examined and found that in the CORN vs. ALFA group, the CORN vs. LEYM group, and the CORN vs. OATS group, 95, 88, 156 metabolites were linked to rumen microbial metabolism. Among them, 44, 70 and 44 metabolites were originated from co-metabolism by the host and microorganisms (Figures 6A–C). Pathway enrichment analysis revealed that 73, 78, and 96 pathways related to microbial metabolism and host-microorganisms’ co-metabolism were annotated in the CORN vs. ALFA group, the CORN vs. LEYM group, and the CORN vs. OATS group, respectively (Figures 6D–F). In the CORN vs. ALFA group, the differential metabolic pathways were associated with host-microorganisms’ co-metabolism included Riboflavin metabolism (ko00740) and Glutathione metabolism (ko00480). The pathway resulting from microbial metabolism identified the flavonoids degradation (ko00946) (*p* < 0.05), with six rumen differential metabolites enriched across these three pathways. In the CORN vs. LEYM group, the pathways related to host-microorganisms co-metabolism encompassed carbon fixation by the calvin cycle (ko00710), arginine biosynthesis (ko00220), beta-alanine metabolism (ko00410), arginine and proline metabolism (ko00330), cyanoamino acid metabolism (ko00460), pantothenate and CoA biosynthesis (ko00770), alanine-aspartate–glutamate metabolism (ko00250), monobactam biosynthesis (ko00261), various antibiotics biosynthesis (ko00998), glutathione metabolism (ko00480) and sphingolipid metabolism (ko00600). The pathways derived from microbial metabolism included arginine biosynthesis (ko00220), amino-benzoate degradation (ko00627), naphthalene degradation (ko00626) and dioxin degradation (ko00621) (*p* < 0.05), with 18 rumen differential metabolites enriched across these 15 pathways. In the CORN vs. OATS group, the differential metabolic pathways related to host-microorganisms’ co-metabolism were histidine metabolism (ko00340), unsaturated fatty acids biosynthesis (ko01040), alpha-linolenic acid metabolism (ko00592), various antibiotics biosynthesis (ko00998), phenylalanine metabolism (ko00360) and glycine, serine, and threonine metabolism (ko00260) (*p* < 0.05), with 31 rumen differential metabolites enriched across these 13 pathways.

**Figure 7:**
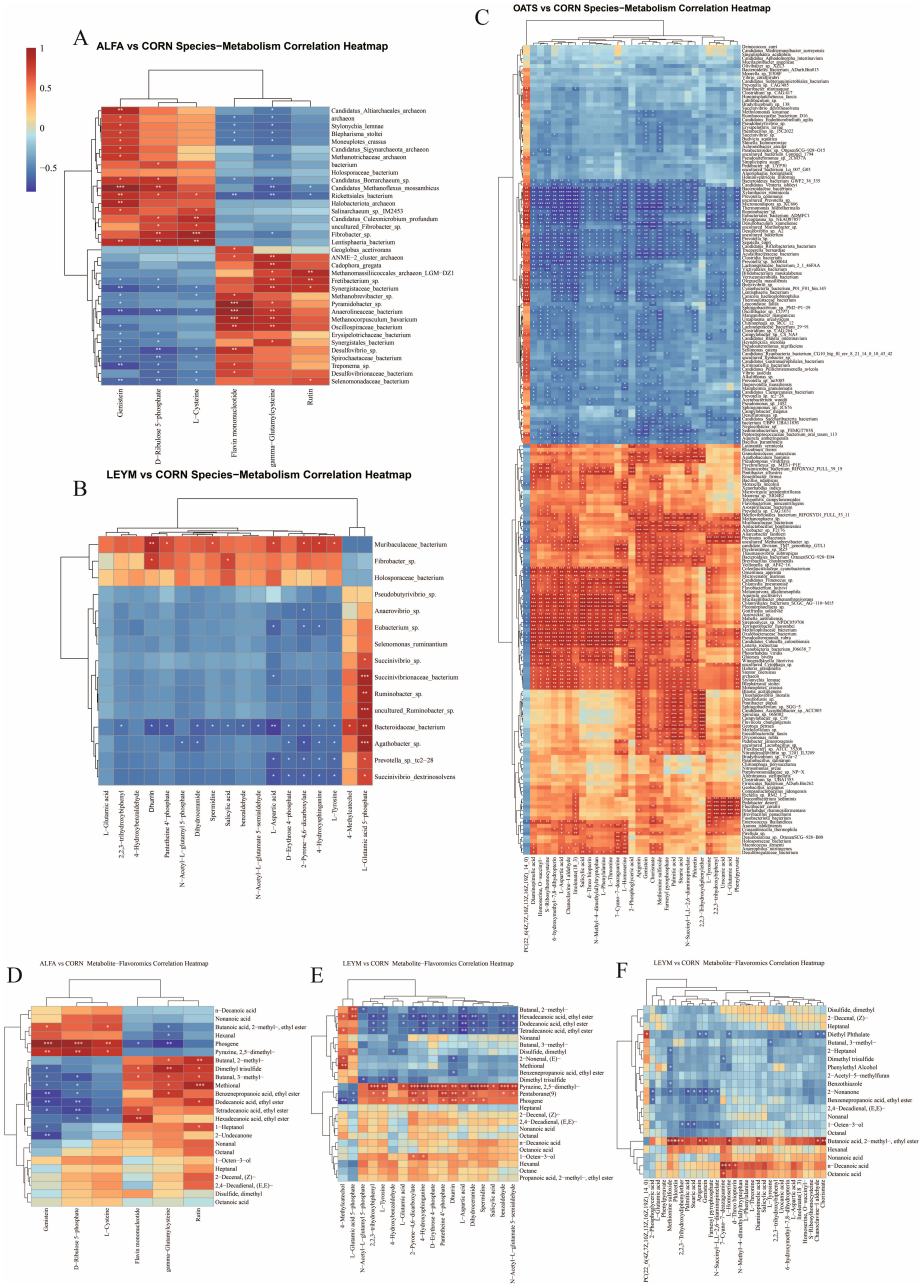
Correlation analysis linking rumen microbiota, metabolites, and muscle flavor compounds. **(A–C)** Pearson correlation heatmaps showing associations between rumen microbial species and rumen metabolites. **(D–F)** Pearson correlation analysis between rumen metabolites and muscle volatile flavor compounds, highlighting key interactions in the microbiota–metabolite–flavor axis.

Pearson correlation analysis at the species level was conducted to further investigate the relationship between rumen microbiota across different forage, corn stalk groups and rumen metabolites enriched in the aforementioned differential pathways like host-microorganisms’ co-metabolism. This analysis focused the different rumen metabolites resulting from microbial metabolism and host-microorganisms’ co-metabolism, in conjunction with the different rumen microorganisms (LDA > 3) within each group, as illustrated in Figures 7A–C. In CORN vs. ALFA groups comparison, D-Ribulose 5-phosphate, L-Cysteine, and Genistein were significantly down-regulated in the ALFA group as compared to CORN group. Conversely, rutin, flavin mononucleotide and gamma-glutamylcysteine exhibited significant upregulation. Notably, gamma-Glutamylcysteine and L-Cysteine are implicated to regulate ko00480, while Flavin mononucleotide and D-Ribulose 5-phosphate regulate ko00740. Additionally, Rutin and Genistein also participate in ko00946 regulation. Certain bacterial species, such as *Oscillospiraceae* sp., demonstrated a significant positive correlation with Flavin mononucleotide and gamma-Glutamylcysteine, while showing a significant negative correlation with Genistein. Bacterial strains like *Selenomonadaceae* sp. and *Synergistaceae* sp. exhibited significant negative correlations with D-Ribulose 5-phosphate, Genistein, and L-Cysteine, but a significant positive correlation with Rutin (*p* < 0.05). The CORN vs. LEYM comparison analysis revealed that metabolites such as L-Aspartic acid and L-Tyrosine were significantly down-regulated in the LEYM as compared to the CORN group. L-Aspartic acid regulated eight pathways (ko00710, ko00220, ko00410, ko00460, ko00770, ko00250, ko00261, ko00998), while L-Tyrosine were involved in two pathways (ko00261, ko00998). Notably, bacterial species such as *Bacteroidaceae* sp. exhibited a significant negative correlation with both L-Aspartic acid and L-Tyrosine. In contrast, bacterial strains like *Muribaculaceae* sp. demonstrated a significant positive correlation with these metabolites (*p* < 0.05). The CORN vs. OATS groups analysis revealed that metabolic substances, including L-Aspartic acid, L-Homoserine, linolenate (18:3), and L-Tyrosine, were significantly down-regulated in the OATS as compared to the CORN group. L-Aspartic acid was associated with three enriched pathways (ko00998, ko00260, ko00340), while L-Glutamic acid regulated two pathways (ko00998, ko00340). L-Homoserine were associated with two pathways (ko00270, ko00300), and linolenate (18:3) participated in the regulation of two pathways (ko01040, ko00592). Additionally, L-Tyrosine is implicated in two pathways (ko00998, ko00360). The bacterial species *Muribaculaceae* sp. exhibited a significant positive correlation with L-Aspartic acid, linolenate (18:3), and L-Tyrosine. Conversely, bacteria such as *Bacteroidaceae* sp., *Prevotella* sp., and *Xylanibacter ruminicola* showed significant negative correlations with L-Aspartic acid, L-Homoserine, linolenate (18:3), and L-Tyrosine (*p* < 0.05).

Pearson correlation analysis was performed to investigate the potential chain effect between rumen metabolites and volatile flavor metabolites in muscle. This analysis compared the differential metabolites from the rumen in CORN vs. ALFA, CORN vs. LEYM, and CORN vs. OATS with different volatile flavor compounds present in the longissimus dorsi muscle, including those associated with the muttony flavor. The results are illustrated in Figures 7D–F. In comparison to the CORN group, the ALFA group exhibited increased levels of 1-Heptanol and 2-Undecanone, associated with a grassy aroma. Additionally, volatile flavor compounds such as Benzenepropanoic acid, ethyl ester, Butanal, 3-methyl- and Dodecanoic acid, ethyl ester, are linked to floral and fruity odors. Conversely, the Pyrazine, 2,5-dimethyl- levels associated with a muttony scent, were down-regulated. Notably, the rumen metabolites D-Ribulose 5-phosphate and Genistein exhibited significant negative correlations with volatile flavor compounds like Benzenepropanoic acid, ethyl ester, Butanal, 3-methyl-, Dodecanoic acid and ethyl ester. Furthermore, the rumen metabolite Genistein also revealed a significant negative correlation with volatile flavor substances like 1-Heptanol and 2-Undecanone. In contrast, the rumen metabolites like D-Ribulose 5-phosphate, Genistein, and L-Cysteine were significantly positively correlated with the muscle volatile flavor compound Pyrazine, 2,5-dimethyl- (*p* < 0.05). Comparing to the CORN group, the LEYM exhibited an up-regulation of muscle volatile flavor compounds like Dimethyl trisulfide and 2-Nonenal,(E)-. Conversely, the pungent-smelling Pentaborane(9) and the mutton-scented Pyrazine were downregulated along with 2,5-dimethyl-. Particularly, rumen metabolites like 4-Hydroxysphinganine, L-Aspartic acid, and L-Tyrosine demonstrated significant positive correlations with volatile flavor substances like Pentaborane(9) and Pyrazine, 2,5-dimethyl-. While the rumen metabolite 4-Methylcatechol showed a significant positive correlation with the muscle volatile flavor compound (E)-2-Nonenal (*p* < 0.05). Comparing the CORN group, the OATS exhibited an up-regulation of 2-Nonanone, Benzenepropanoic acid, ethyl ester, 2-Heptanol, and Phenylethyl Alcohol, associating to fruity and floral odors. Conversely, Octanoic acid and n-Decanoic acid, linked to the characteristic muttony smell, were found to be reduced. Notably, the muscle volatile flavor compounds like 2-Heptanol and Phenylethyl alcohol revealed a significant negative correlation with the rumen metabolite Methionine sulfoxide. Additionally, the muscle volatile flavor compound 2-Nonanone was also significantly negatively correlated with rumen metabolites such as Palmitic acid and Stearic acid. The muscle volatile flavor compounds like Benzenepropanoic acid and ethyl ester also exhibited significant negative correlations with the rumen metabolites such as 2-Phosphoglyceric acid and Genistein. Among the rumen metabolites, 7-Cyano-7-deazaguanine showed a significant positive correlation with Octanoic acid and n-Decanoic acid, but was significantly negatively correlated with Dimethyl trisulfide. Furthermore, the rumen metabolites like d-Threo biopterin and L-Homoserine were significantly positively correlated with n-Decanoic acid (*p* < 0.05).

## Discussion

Mutton constitutes an important component of the global meat supply, contributing extensively in red meat production. Globally, red meat estimates approximately 26% of total meat production of which mutton accounts nearly 11% (38). Mutton quality (physicochemical and sensory) is regulated by various factors, including the host lipid and amino acid metabolism, microbial metabolism, dietary nutrition and the forage type consumption. This practice not only significantly upgrades the weight gain, dry matter intake and feed conversion efficiency of lambs, but also these dietary approaches enhance the quality attributes of meat by decreasing the drip and cooking loss, as well as increasing the color stability and the overall sensory properties (39–42). In this study, the provision of high-quality forage (LEYM, OATS, ALFA) significantly elevated the average daily feed intake (ADFI) and average daily weight gain (ADG) of lambs compared to the CORN group. Additionally, the ALFA group exhibited a reduced feed conversion ratio (F/G) due to its higher crude protein content, promoting rumen microbial protein synthesis. Furthermore, its low lignin content enhanced digestibility and improved the energy-protein conversion efficiency (43). The OATS group was linked with reduced cooking loss of mutton due to improved muscle water-holding capacity, increased muscle fiber density and enhanced metabolic efficiency, thus contributing towards superior meat tenderness (44, 45). The LEYM group showed reduced water loss and muscle yellowness (b* value) at 30 min’ post-slaughter. These effects may be linked with enhanced intramuscular fat deposition by modulating fatty acid metabolism, which lowers the b* value and improves meat quality and as well as with bioactive compounds like *α*-tocopherols and polyphenolic which inhibit lipid and protein oxidation during thermal process (46). This process slows the degeneration and contraction of myofibrils, reduces free water expulsion, and consequently decreases cooking loss (47). These results demonstrate feeding of high-quality forage can improve the production performance and meat quality of lambs in different dimensions.

The rumen microbial community and associated metabolites, known as regulators, mediate the diet-effects on meat quality (48). The important roles of gastrointestinal microbiota and lipid metabolism in the formation of flavor precursors; provide insights to improve lamb flavor through the management of microbial populations and metabolic processes (49). Some previous studies have investigated the regulatory effects of corn stalks, sheep grass hay, alfalfa and oat grass on growth performance, carcass traits and meat flavor of ruminants (40, 50, 51). However, several knowledge gaps persist in application of multi-omics studies that systematically elucidate the mechanisms linking different forages to rumen microorganisms, metabolites, and meat flavor (52). By using corn stalks as a control, previous work systematically revealed the association between rumen microbiota and metabolite composition and volatile flavor substances in the lambs’ muscles fed with different high-quality forage, intending to provide a theoretical basis for regulating the muscle flavor of ruminants through forage (53).

The rumen microbiota composition constitutes a central determinant of fermentation patterns, with resultant fermentation metabolites affecting the host muscle characteristics (54). In this study, *Firmicutes* and *Bacteroidetes* were identified as the most prevalent bacterial phyla, consistent with previous findings (55, 56). Methanobacteriota were reported highly abundant in the archaeal community, possibly due to its ability of using hydrogen (H_2_) produced by anaerobic fungi, fascilitating methanogenesis and enhancing the fiber degradation efficiency (57). Ciliophora was appeared as the dominant phylum among eukaryotic microorganisms, and plays a role in rumen homeostasis by stabilizing pH and anaerobic conditions (58). The differential abundance based LEfSe analysis indicated the different microbial signatures of dietary treatments. Compared to the CORN group, the ALFA group exhibited an increased relative abundance of bacterial species like *Scillospiraceae*, *Selenomonadaceae* and *Synergistaceae*. These microbial taxa have previously been associated with the production of short-chain fatty acids (SCFAs) (59), the improvement of feed conversion rates (60) and the reduction of plant toxicity (61). The LEYM group showed an increase in the abundance of *Succinivibrio* sp. and *Bacteroidaceae* sp., alongside a decrease in *Muribaculaceae* sp. This compositional shift promotes the conversion of rumen succinic acid to propionic acid, thereby enhancing rumen energy metabolism and cellulose degradation (62, 63). The OATS group was recognized by relative abundance of *Xylanibacter ruminicola*, *Bacteroidaceae* sp., *Kiritimatiellia* sp., *Clostridia* sp., and *Prevotella* sp. This enhanced abundance facilitates rumen cellulose degradation and improves the conversion efficiency of roughage (64, 65).

The gastrointestinal microbiota of rumen, particularly within the rumen and posterior digestive tract, plays a defining role in bioconversion of dietary carbohydrates, proteins and lipids into diverse range of intermediate metabolites (32). These metabolites then participate in complex biochemical pathway, which develop the meat flavor, ultimately contributing to the distinctive sensory attributes of lamb (66). Among them, 3-methylindole (associated with fecal odor) and 4-methylphenol (p-cresol), produce an undesirable fecal odor. These metabolites are produced through microbial-based transamination and decarboxylation of amino acids and may accumulate in the adipose tissue of sheep, thereby enhancing the mutton like flavor of sheep meat (67). Free amino acids and dipeptides serve as crucial flavor precursors in meat (68). Additionally, aromatic amino acids like tyrosine and tryptophan, significantly influence the formation of “pastoral flavor” compounds. Notably, tryptophan and tyrosine, produce 3-methylindole and 4-methylphenol, respectively (69). While Alpha-linolenic acid serves as a significant precursor of muttony substances and its abundance is positively correlated with the mutton-like smell intensity in the longest muscle of sheep’s back and the levels of stearic acid and oleic acid. In addition, the metabolic interactions between stearic acid, oleic acid, and 0-linolenic acid coordinate the regulation of fatty acid biosynthetic and oxidation systems, further affecting flavors outcomes (70). In the analysis of rumen metabolites in the LEYM group, tyrosine and linolenic acid were significantly downregulated compared to the CORN group. Additionally, the OATS group exhibited significant down-regulation of tyrosine, tryptophan, stearic acid, linolenic acid and 4-methylphenol relative to the CORN group. Finally, the precursors associated with mutton-like odor in the lambs’ rumen feeding LEYM and OATS groups were highly down-regulated relative to the CORN group. Consequently, the mutton-like odor resulting from high-quality forage consumption was improved to some extent compared to feeding corn stalks.

Volatile flavor compounds are critical parameters for assessing meat quality. Fatty acids like octanoic acid, 2-methyloctanoic acid, nonanoic acid, nonenoic acid, and 8-methylnonanoic acid may contribute to production of mutton odor (71). Pyrazine, a heterocyclic compound with a potent aroma, are closely associated with the mutton-like smell (72). Analyzing volatile flavor substances in muscle tissues, 2,5-dimethyl- was significantly down-regulated when compared to fatty acids like octanoic acid, nonanoic acid, nonenoic acid, decanoic acid while pyrazine in both the CORN and ALFA groups. The marked down-regulation of these fatty acids along with 2,5-dimethyl-, were also observed in the LEYM group. The levels of fatty acids like octanoic acid, nonanoic acid, decanoic acid, and 8-methylnonanoic acid in the OATS group were significantly reduced (*p* < 0.05) (73). The compounds like dimethyltrisulfide, 3-methylbutanal, and (E)-2-nonenal were identified as key aromatic constituents in thyme-stewed mutton, enhancing flavor characteristics. In this study, dimethyltrisulfide and 3-methylbutyraldehyde levels were significantly upregulated (*p* < 0.05) in the group fed with high-quality forage compared to the CORN group. Additionally, volatile organic compounds (VOCs), including aldehydes, ketones, esters, and alkanes, play a crucial role in shaping the aroma of mutton. For example, elevated concentrations of ethanol exhibit flavors reminiscent of vanilla, wood and high fat to meat products (74). Ketones contribute floral, fruity and creamy aromas (75), while ethyl acetate adds fruity and wine-like fragrances that enhance the mutton flavor (76). Furthermore, the ALFA, LEYM, and OATS forage feeding groups demonstrated significant up-regulation (*p* < 0.05) of aldehydes (e.g., butanal, 2-methyl-, methional), ketone bodies (e.g., 2-nonanone), esters (e.g., hexadecanoic acid ethyl ester, benzenepropanoic acid ethyl ester), and alkanes (e.g., 2-undecanone) when compared to the CORN group. Therefore, feeding high-quality forage, in contrast to low-quality coarse feed such as corn stalks, can effectively attenuate the undesirable mutton-like odor while improving favorable aroma attributes. A multi-omics correlation analysis was performed to investigate the mechanisms by which high-quality forage influences rumen metabolites and subsequently affects muscle flavor through alterations in rumen microbiota composition. Compared to the volatile flavor compounds present in the longissimus dorsi muscle of the CORN group, the ALFA group exhibited an up-regulation of 1-Heptanol, denoted by a grassy odor and 2-Undecanone associated with orange, fresh and green aromas. Conversely, the mutton-related compound Pyrazine and 2,5-dimethyl-, was down-regulated. The sheep rumen environment fed with the ALFA diet, a decrease in the abundance of archaeal species and specific bacteria, including *Oscillospiraceae, Selenomonadaceae*, and *Synergistaceae*, was observed. This microbial shift may be associated with the down-regulation of Genistein and the upregulation of Rutin via the ko00946 pathway. Consequently, the increased level of 1-Heptanol and 2-Undecanone also contributes to the down-regulation of D-Ribulose 5-phosphate and L-Cysteine through ko00740 and ko00480 pathways, ultimately, down-regulating the Pyrazine, 2,5-dimethyl- in the muscle. As compared to the volatile flavor compounds in the longissimus dorsi muscle of the CORN group, the LEYM group showed an upregulation of 2-nonenal (E), having a waxy and cucumber-like odor, alongside a downregulation of Pentaborane(9), known for its pungent scent, and Pyrazine, 2,5-dimethyl-, linked to the mutton flavor.

**Figure 8:**
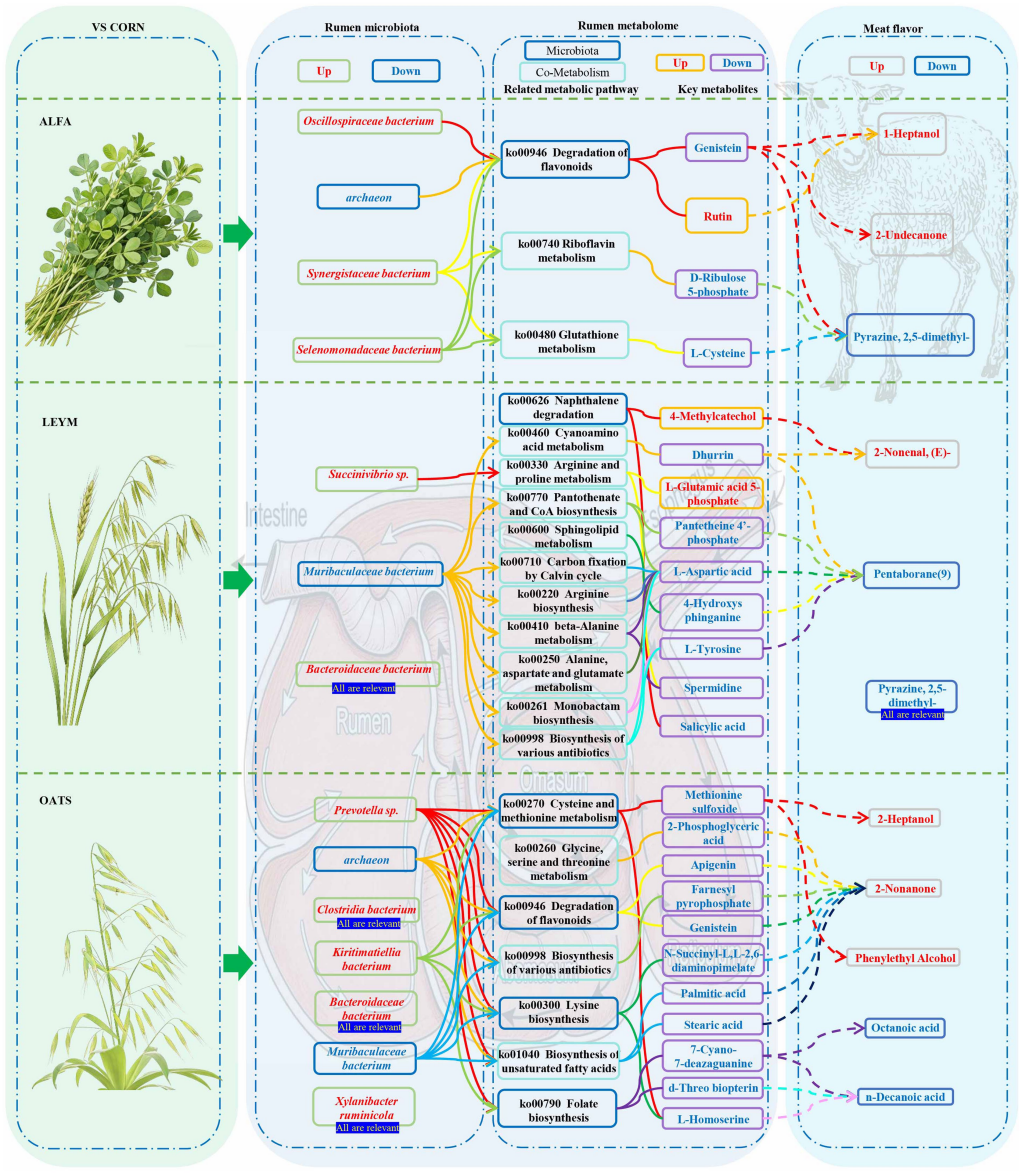
Integrated schematic model illustrating the effects of high-quality forage on rumen microbiota, metabolism, and meat flavor formation. This figure summarizes the proposed mechanism by which dietary forage modulates rumen microbial composition, alters metabolite profiles, and subsequently influences muscle volatile flavor compounds. The model highlights key microbial taxa, metabolic pathways, and flavor-related metabolites involved in the “forage–microbiota–metabolism–flavor” regulatory axis.

The rumen of LEYM group sheep showed an increase in the abundance of *Succinivibrio* sp. and *Bacteroidaceae* sp. and a decrease in the abundance of *Muribaculaceae* sp. indicating a change in microbial composition due to diet. This alteration could have favoured the up-regulation of 4-Methylcatechol via the ko00626 pathway and the down-regulation of Dhurrin through the ko00460 pathway, ultimately influencing the up-regulation of 2-Nonenal (E) in the muscle. Additionally, L-Glutamic acid 5-phosphate was upregulated through the ko00330 pathway, while Pantetheine was also upregulated via the same pathway. Conversely, 4′-phosphate was downregulated, and L-Aspartic acid was released through multiple pathways, including ko00710 and ko00770. Furthermore, 4-Hydroxysphinganine was downregulated through the ko00600 pathway, while L-Tyrosine was downregulated via the ko00261 and ko00998 pathways, ultimately affecting Pentaborane(9) and Pyrazine in the muscle. The downregulation of 2,5-dimethyl- is illustrated in Figure 8.

The OATS group also displayed a significant up-regulation of the desirable volatile compounds in the longissimus dorsi muscle compared to the CORN group; these desirable volatile compounds included 2-heptanol, phenylethyl alcohol and 2-nonanone, which give rise to fruity and floral fragrances. Conversely, major odor-forming fatty acids including octanoic acid and n-decanoic acid were greatly suppressed, which means that the mutton-like odor was decreased. The rumen of OAT group sheep increased the abundance of *Prevotella* sp., *Clostridia* sp., *Kiritimatiellia* sp., *Bacteroidaceae* sp., and *Xylanibacter ruminicola* strains. Possibly, the ko00270 pathway downregulates Methionine sulfoxide, thereby influencing the upregulation of 2-Heptanol, 2-Nonanone, and Phenylethyl alcohol in the muscle. Additionally, this pathway may downregulate 2-Phosphoglyceric acid via ko00260, as well as Apigenin and Genistein through ko00946. Moreover, the concerted depletion of intermediates like 2-phosphoglyceric acid (ko00260), apigenin and genistein (ko00946), farnesyl pyrophosphate (ko00998) and N-succinyl-L, L-2,6-diaminopimelate (ko00300) may collectively contribute to the enhancement of desirable flavor compounds, especially 2-nonanone. The relative abundances of *Prevotella* sp., *Clostridia* sp., *Kiritimatiellia* sp., *Bacteroidaceae* sp., and *Xylanibacter ruminicola* strains was also increased. Moreover, the reduction of metabolites 7-cyano-7-deazaguanine and d-threo-biopterin via the ko00790 pathway and the down-regulation of L-homoserine by ko00270 may be mechanistically associated with down-regulated production of octanoic acid and n-decanoic acid in muscle tissue (Figure 8). There were strong associations between forage type, rumen microbiota, metabolites, and muscle volatile compounds, but the current results are correlative. Hence, additional mechanism and functional validation studies are needed to identify direct causal relationships. Microbota–metabolite–flavor axis was confirmed by the integrated changes at the microbial, rumen metabolic and volatile muscle level under the various forage treatments. MetOrigin analysis indicated several differential metabolites related to microbial or host–microbial co-metabolic pathways, indicating potential associations between rumen microorganisms and biotransformation of metabolites. Based on the KEGG classification, several enriched pathways identified in the present study were classified as environmental or xenobiotic degradation pathways. These annotations should not be used to indicate exposure to any environmental pollutants. All of the rumen microbial enzymes that transform plant secondary metabolites, metabolize aromatic compounds and bio-transform lipids have common enzymatic reactions and intermediates with the KEGG environmental degradation pathways, which is why they are often shared. So, the enrichment of these pathways is probably the consequence of the diverse metabolic capabilities of the rumen microorganisms to degrade the organic compounds present in complex forage. Moreover, strong associations between certain microbial taxa and metabolites and volatile compounds further indicated the presence of associative relationships between forage–rumen–muscle flavor. All these relationships are correlative and hypothesis-generating, however, and future mechanistic studies are needed to confirm direct causal interactions.

One of the limitations of the present study includes validation of the flavor characterization, which was not carried out by trained sensory panels or consumer sensory evaluation using quantification of the volatiles and relative odor activity value (ROAV). Thus, the identified odor characteristics should be regarded as predictive characteristics rather than sensory characteristics, according to the composition of volatile compounds. Further research involving a trained sensory panel evaluation and incorporation of flavor omics will give a more comprehensive validation of the changes in flavor perception of lamb meat in relation to forage.

## Conclusion

This research paper demonstrates that the substitution of low-quality corn stalks by the high-quality forages like alfalfa (ALFA), *Leymus chinensis* (LEYM) and oat hay (OATS), significantly improves both the growth performance and meat quality of lambs. Increased average daily feed intake and weight gain, coupled with positive meat quality characteristics, were observed like less cooking loss (OATS) and less drip loss and yellowness (LEYM). This work contributes towards the understanding of the biological processes involved in improving mutton flavor through an integrated multi-omics approach. Particularly, the forage quality was associated with alterations in the rumen microbial community and corresponding microbial or host-microbial metabolic pathways. These changes controlled the important rumen metabolites, which in turn were associated with changes in volatile flavor compounds in muscle along the forage-rumen microbiota-metabolite-muscle axis. Notably, ALFA, LEYM, and OATS dietary supplementation inhibited the formation of undesirable mutton flavor precursors (e.g., aromatic amino acids include tryptophan and tyrosine) and facilitated the formation of desirable flavor compounds. This was indicated by decreased levels of odor related compounds like octanoic acid, nonanoic acid, pyrazine derivatives and increased levels of aldehydes, ketones, and esters with fruity, floral and pleasant flavors. Altogether, these findings support a mechanistic model that connects the forage quality with the meat flavor through rumen microbial and metabolic control. These observations offer scientific basis for precision nutritional options aimed at enhancing the flavor and consumer acceptability of mutton. Nevertheless, although strong correlations were observed, and further specific investigations are required to prove the cause-effect relationships of certain microbial taxa, metabolic pathways and the formation of flavor compounds.

## Data Availability

The original contributions presented in the study are included in the article/Supplementary material, further inquiries can be directed to the corresponding authors.

## Footnotes

1
https://www.biodeep.cn

## Supplementary material

Supplementary Figure 1 OPLS-DA analysis of rumen metabolites under different dietary treatments. **(A–C)** OPLS-DA score plots under positive ion mode. **(D–F)** Validation plots of OPLS-DA models under positive ion mode. **(G–I)** OPLS-DA score plots under negative ion mode. **(J–L)** Validation plots of OPLS-DA models under negative ion mode.

Supplementary Figure 2 Multivariate analysis and clustering of differential metabolites. **(A–C)** OPLS-DA score plots of differential metabolites. **(D–F)** Permutation test validation of OPLS-DA models. **(G–I)** Heatmaps showing clustering of the top 50 differential metabolites.
