# Supplementary material for: An integrative multi-omics investigation into the influence of forage type on the volatile flavor profile of Ujumqin sheep mutton
Source: Front Vet Sci. 2026 Jun 16;13:1856240. doi: 10.3389/fvets.2026.1856240 (PMC13314528; doi:10.3389/fvets.2026.1856240)
Supplement: Supplementary file 3 [file Table_1.DOCX]

**Supplementary Table S1.** The alpha diversity index of the rumen microbiota.

| Sample | ALFA | LEYM | OATS | CORN | *P*-value |
| --- | --- | --- | --- | --- | --- |
| simpson_AVG | 0.071783333 | 0.089783333 | 0.096283333 | 0.0798 | 0.0950717872315589 |
| simpson_SD | 0.019012566 | 0.017723816 | 0.013218989 | 0.009087574 |  |
| chao1_AVG | 8902.65 | 8841.666667 | 8849.983333 | 8665 | 0.470491090470402 |
| chao1_SD | 303.4471338 | 487.1842595 | 325.6026868 | 237.8097475 |  |

**Supplementary Table S2.** ROAV data integration table.

| name | Class | Rangeof  OdorMin | Rangeof  OdorMax | Odor  Character | CORN  ROAV | LEYM  ROAV | OATS  ROAV | ALFA  ROAV |
| --- | --- | --- | --- | --- | --- | --- | --- | --- |
| Dodecanoic acid, ethyl ester | Esters | 500 |  | Sweet, Waxy, Floral | 7.1424E-05 | 4.96689E-05 | 4.03829E-05 | 6.11229E-05 |
| 2-Propanol, 1-methoxy- | Alcohols | 8390 | 33000 | etherish, ammonia | 1.42376E-06 | 3.59387E-07 | 1.50917E-07 | 1.76532E-07 |
| Octane | Hydrocarbons | 660 | 235000 | gasoline, oil | 3.18153E-05 | 2.4685E-06 | 2.5756E-07 | 9.18613E-07 |
| 2-Nonenal, (E)- | Aldehydes | 0.0002 |  | Fatty, Cucumber | 82.25597978 | 85.0191164 | 100 | 87.38787782 |
| Benzenepropanoic acid, ethyl ester | Esters | 130 |  | Floral, Rosy-Flavor, Fruity | 3.81175E-06 | 5.29555E-06 | 3.59442E-06 | 5.62354E-06 |
| Methional | Aldehydes | 7.12 |  | Cooked Potato | 0.001211297 | 0.001156513 | 0.000642495 | 0.000880671 |
| 2-Heptanol | Alcohols | 200 |  | Fruity, Mouldy, Musty, Mushroom | 2.47884E-07 | 7.18938E-07 | 5.63578E-09 | 5.69517E-08 |
| Butanoic acid, 2-methyl-, ethyl ester | Esters | 1.6 |  | Fruity | 0.000405775 | 0 | 9.4667E-06 | 1.22227E-05 |
| Phosgene |  | 120 | 5700 | hay like | 9.80856E-06 | 3.57313E-06 | 4.61355E-07 | 0 |
| Diethyl Phthalate | Esters | 36 | 363 |  | 0 | 4.92176E-06 | 0 | 5.88298E-07 |
| Chlorine dioxide | Homogeneous non-metal compounds | 15000 |  | chlorine | 0 | 4.88326E-09 | 0 | 0 |
| Pentanoic acid, 2-hydroxy-4-methyl-, ethyl ester | Esters | 126 |  | Blackberry | 2.27227E-05 | 0 | 1.77667401794924E-07 | 2.37732E-07 |
| 2(3H)-Furanone, 5-heptyldihydro- | Organoheterocyclic compounds | 4.2 |  |  | 3.42409E-05 | 2.26051E-05 | 1.66763E-05 | 2.31089E-05 |
| 1-Hexanol, 2-ethyl- | Alcohols | 0.198 |  | Rose, Green | 0.007045286 | 0.018463043 | 0.002570081 | 0.000670114 |
| Propanoic acid, ethyl ester | Esters | 19019.33 |  | Ruity, Banana-Flavor | 9.1619E-07 | 2.98512E-07 | 1.73784E-07 | 1.57831E-07 |
| Butanoic acid, ethyl ester | Esters | 9.5 |  | Fruity | 0.002688576 | 0.000659123 | 0.000837536 | 0.000380148 |
| Ethane, 1,1-diethoxy- | Ethers | 719 |  | Fruity | 2.63003E-06 | 1.88622E-06 | 7.65046E-07 | 6.89312E-07 |
| Caprolactam | Organoheterocyclic compounds | 65 |  | mild | 5.40503E-07 | 0 | 8.76511E-08 | 8.11366E-08 |
| Heptanoic acid, ethyl ester | Esters | 13153.17 |  | Fruity | 7.81988E-06 | 5.20687E-06 | 2.82361E-06 | 3.45588E-06 |
| Octanoic acid, ethyl ester | Organoheterocyclic compounds | 147 |  | Fruity | 0.001376024 | 0.000488449 | 0.000238495 | 0.000283557 |
| p-Cresol | Benzenoids | 60 |  |  | 4.48679E-05 | 2.64724E-05 | 1.37569E-05 | 2.89241E-05 |
| Benzene, 1,4-dichloro- | Benzenoids | 121 | 15000 | camphor, mothballs | 5.35748E-05 | 2.00831E-05 | 1.31632E-05 | 1.27978E-05 |
| Hexanoic acid, methyl ester | Esters | 87 |  | Floral, Fruity | 3.65361E-07 | 3.53645E-06 | 1.17958E-07 | 6.14388E-08 |
| 2-Propenal | Organic oxygen compounds | 3.6 | 1800 | pungent | 0 | 0 | 8.94461E-05 | 6.52304E-05 |
| 2-Pentanone | Ketones | 28 | 65000 | fingernail polish | 6.02559E-05 | 2.14409E-05 | 1.79581E-05 | 7.27918E-05 |
| Propane, 1-nitro- | Organic 1,3-dipolar compounds | 7700 | 140000 |  | 0 | 2.95667E-09 | 0 | 0 |
| Acetic anhydride | Carboxylic_Acids | 120 | 360 | sour, acid | 0 | 2.52618E-07 | 0 | 0 |
| Maleic anhydride | Organoheterocyclic compounds | 250 | 320 | acrid | 5.8455E-08 | 0 | 0 | 0 |
| Phenol, 3-methyl- | Benzenoids | 20 |  | Smoky,Petroleum-Like | 0 | 0 | 0 | 3.52451E-06 |
| Pyrazine, 2,6-dimethyl- | Organoheterocyclic compounds | 790.79 |  | Green Pepper Fragrant | 4.44488E-06 | 2.31236E-06 | 4.57321E-07 | 8.21293E-07 |
| Butanoic acid, 3-methyl-, ethyl ester | Esters | 6.89 |  | Fruity, Apple-Flavor | 0 | 0 | 2.20137E-07 | 0 |
| Cyclohexanone | Ketones | 52 | 219000 | sweet, sharp | 4.20869E-05 | 6.18952E-06 | 2.22976E-06 | 5.50251E-06 |
| Phenol | Benzenoids | 4.5 | 1950 | medicinal, acid,ink, creosote,empyreumatic | 0 | 1.52853E-06 | 0 | 0 |
| Pyridine, 2-methyl- | Organoheterocyclic compounds | 2.6 | 23.6 | strong, unpleasant | 7.23346E-05 | 2.11254E-05 | 5.78619E-06 | 1.40491E-05 |
| Pyrazine, methyl- | Organoheterocyclic compounds | 121927.01 |  | Toasted Bread, Roasted Almonds, Fried Peanuts | 1.19966E-08 | 6.89902E-09 | 4.14246E-09 | 4.0514E-09 |
| Ethyl formate | Esters | 2700 | 30000 | aromatic | 8.00463E-06 | 4.59527E-06 | 2.42307E-06 | 2.96372E-06 |
| Thiophene | Organoheterocyclic compounds | 511 |  | Garlic, Alliaceous | 2.56217E-07 | 2.37144E-07 | 1.3206E-07 | 1.78984E-07 |
| Decanoic acid, ethyl ester | Esters | 420 |  | Brandy | 0.000732599 | 0.000223243 | 0.000135762 | 0.000206241 |
| 2-Heptanone | Ketones | 0.75 | 710 | sweet, mushroom | 0.011816095 | 0.027185114 | 0.003578774 | 0.010252101 |
| Pentanal | Aldehydes | 0.4 | 4970 | sickening, rancid,decayed | 0.302265339 | 0.19315435 | 0.078544342 | 0.102576388 |
| Cyclohexene | Hydrocarbons | 180 |  | sweet | 0 | 0 | 0 | 1.16825E-08 |
| Pyridine | Organoheterocyclic compounds | 10 | 12000 | burnt, pungent,nauseating | 0.000150802 | 9.01484E-05 | 3.21816E-05 | 4.55244E-05 |
| 5-Hepten-2-one, 6-methyl- | Ketones | 50 |  | Herby, Green, Citrus, Musty, Lemongrass | 2.08106E-05 | 2.59745E-05 | 6.56059E-06 | 8.75118E-06 |
| Octanoic acid, methyl ester | Esters | 200 |  | Fruity, Citrus-Like | 0 | 2.24346E-07 | 0 | 0 |
| 1-Hexanol | Alcohols | 2.4 | 16000 | green grass, plastic | 0.044393146 | 0.032815766 | 0.014902415 | 0.019742374 |
| Glutaraldehyde | Aldehydes | 0.37 | 39 |  | 0 | 0 | 0 | 0.000130414 |
| 1-Hexanethiol |  | 0.39 |  | Baked Taste | 0 | 0 | 0 | 2.42417E-05 |
| 1-Octene | Hydrocarbons | 1 | 206000 |  | 0.001645845 | 2.73994E-05 | 0.000404022 | 0.000343363 |
| 1-Heptanol | Alcohols | 3 |  | Grassy | 0.013411637 | 0.012492697 | 0.006178636 | 0.007652691 |
| Heptanal | Aldehydes | 0.003 |  | Citrus, Fatty, Rancid | 64.57717462 | 45.64487362 | 17.25772437 | 22.38105936 |
| Ethanol, 2-butoxy- | Ethers | 80 | 350 | sweet, ester, musty | 3.46634E-07 | 0 | 2.32416E-07 | 0 |
| Nonane | Hydrocarbons | 2300 | 21000 | gasoline | 1.35731E-07 | 0 | 0 | 0 |
| 1-Octanol | Alcohols | 0.9 | 1690 | penetrating | 0.068869467 | 0.070126212 | 0.029317038 | 0.035131718 |
| Nonanoic acid | Lipids and lipid-like molecules | 3559.23 |  | Fat Smell | 1.49822E-05 | 6.27209E-06 | 4.3277E-06 | 3.38103E-06 |
| 2-Undecanone | Ketones | 0.004355 |  | Orange, Fresh, Green | 0.244956708 | 0.214986501 | 0.145146716 | 0.158577549 |
| Pyrazine, tetramethyl- | Organoheterocyclic compounds | 80073.16 |  | Sweet, Fruity, Floral, Peach | 8.4944E-10 | 4.81794E-10 | 3.27839E-10 | 1.5087E-09 |
| Propene | Hydrocarbons | 10100 | 99000 | gassy, aromatic | 8.64115E-09 | 2.82185E-09 | 8.99983E-09 | 0 |
| 2-Acetyl-5-methylfuran | Ketones | 40870.06 |  | Biscuits, Toasted Almonds, Soap | 1.6291E-09 | 4.42233E-09 | 5.66812E-09 | 2.42589E-09 |
| Triethylamine | Heterocyclic_Compounds | 5 | 2900 | fishy, amine | 1.21769E-05 | 0 | 0 | 0 |
| Phenol, 4-ethyl- | Benzenoids | 440 |  | Phenolic, Leather | 0 | 3.15219E-08 | 0 | 0 |
| Butanedioic acid, diethyl ester | Esters | 353000 |  | Fruity, Sweet | 1.78817E-08 | 1.45071E-08 | 5.61865E-09 | 8.56227E-09 |
| Nonanoic acid, ethyl ester | Esters | 3150.61 |  | Floral, Fruity | 5.33036E-05 | 3.70028E-05 | 1.63672E-05 | 1.84248E-05 |
| Pyrazine, 2,5-dimethyl- | Organoheterocyclic compounds | 3201.9 |  | Green Grass, Fried Bean Spice | 2.12496E-07 | 6.36143E-08 | 1.5314E-09 | 5.40507E-09 |
| beta-Myrcene | Lipids and lipid-like molecules | 773 |  | Geranium | 3.20464E-07 | 3.58532E-07 | 1.75878E-07 | 2.0095E-07 |
| Propanal | Aldehydes | 1 | 101000 | fruity | 0.041896247 | 0.015591476 | 0.006365283 | 0.007158617 |
| Hexanoic acid, ethyl ester | Esters | 30 |  | Fruity, Green Apple | 0.01025784 | 0.004274018 | 0.001347154 | 0.001886423 |
| Butanal | Aldehydes | 0.3 | 5090 | pungent | 0.045866735 | 0.016136732 | 0.006212085 | 0.010120781 |
| Tetradecanoic acid, ethyl ester | Esters | 500 |  | Sweet, Waxy | 5.44386E-05 | 3.63126E-05 | 3.37964E-05 | 3.97217E-05 |
| Octanoic acid | Lipids and lipid-like molecules | 500 |  | Rancid, Cheese, Fatty Acid | 2.51845E-05 | 9.75065E-06 | 9.20641E-06 | 8.04955E-06 |
| Octanal | Aldehydes | 2.5 |  | Lemon, Citrus, Green Grass | 0.046718309 | 0.036229323 | 0.015873695 | 0.017880711 |
| Nonanal | Aldehydes | 1 |  | Aldehyde, Citrus, Orange Peel | 0.17467798 | 0.162485072 | 0.064357545 | 0.078739327 |
| Carbon dioxide |  | 39000000 | 600136000 |  | 3.99061E-10 | 5.85243E-11 | 1.08716E-10 | 1.1138E-10 |
| Tetrachloroethylene | Organohalogen compounds | 767 | 71000 | etherish | 5.25673E-10 | 0 | 5.70664E-09 | 0 |
| 5-Methyl-2-thiophenecarboxaldehyde | Organoheterocyclic compounds | 275 |  | Cherry, Swee | 3.06145E-06 | 2.14568E-06 | 3.98414E-08 | 1.50431E-07 |
| Limonene | Lipids and lipid-like molecules | 1.8 | 310 | lemon, plastic, citrus,rubber, terpeny | 0 | 0.000100831 | 4.04648E-05 | 0 |
| 2-Propenoic acid, ethyl ester | Esters | 0.0066 | 3.2 | sweet,ester,plastic, alcohol, sharp,ammoniacal | 0.074358157 | 0.006687127 | 0.034858267 | 0.00574014 |
| Ethyl Acetate | Esters | 90 | 190000 | fruity, sweet,fingernail polish,etherous | 0.001015177 | 0.000853512 | 0.000542253 | 0.000592213 |
| 3-Penten-2-one, 4-methyl- | Organic oxygen compounds | 17 | 12000 | sweet | 0 | 5.55162E-06 | 0 | 0 |
| Hexanoic acid | Lipids and lipid-like molecules | 3000 |  | Roses, Geraniumc | 1.13042E-05 | 7.05939E-06 | 3.20549E-06 | 4.64874E-06 |
| Dodecanoic acid | Lipids and lipid-like molecules | 9153.79 |  | Greasy, Slightly, Pine, Wood | 2.19141E-07 | 9.00848E-08 | 8.24986E-08 | 1.02351E-07 |
| Pyrazine, trimethyl- | Organoheterocyclic compounds | 0.023 |  | Roasted Nuts, Cocoa, Peanuts | 0.001646718 | 0.009638836 | 0.004495169 | 0.022588498 |
| Pentaborane(9) | Benzenoids | 970 |  | pungent | 1.17731E-07 | 3.40788E-08 | 7.29827E-09 | 9.43156E-08 |
| 2-Dodecenal, (E)- | Aldehydes | 0.00053 |  | Green, Fatty, Sweet | 0.342340102 | 0.260744275 | 0.069716599 | 0.092563765 |
| gamma-Dodecalactone |  | 7 |  | Slight Coconut, Fruity | 0.000135773 | 5.80215E-05 | 3.91275E-05 | 4.10439E-05 |
| 2,4-Decadienal, (E,E)- | Aldehydes | 2.9 |  | Dusty, Waxy, Oily, Soapy | 0.003926118 | 0.004236159 | 0.000534555 | 0.000915594 |
| Pentanoic acid, 4-methyl-, ethyl ester | Esters | 6 |  | Fruity | 0.00014367 | 4.64936E-05 | 2.0449E-05 | 8.15585E-06 |
| 2-Octenal, (E)- | Aldehydes | 0.003 |  | Nuts, Green, Fatty | 13.33576289 | 8.51571472 | 2.653663874 | 3.745644028 |
| Thiazole | Organoheterocyclic compounds | 740 |  | Nut, Sulfur, Stink | 7.63742E-07 | 5.58318E-07 | 2.71353E-07 | 2.59472E-07 |
| Furan, 2-ethyl- | Organoheterocyclic compounds | 2.3 |  | Burnt,Sweet,Coffee-Like | 0.009000005 | 0.004596161 | 0.001608145 | 0.002050662 |
| n-Decanoic acid | Lipids and lipid-like molecules | 15000 |  | Fatty, Rancid, Soap | 5.99599E-07 | 1.17968E-07 | 1.36444E-07 | 1.21846E-07 |
| 1-Octen-3-ol | Alcohols | 11 |  | Mushroom | 0.010631689 | 0.009130463 | 0.001794084 | 0.002306089 |
| Dimethyl trisulfide | Organosulfur compounds | 0.36 |  | Sulfurous,Onion, Spicy | 0.000955183 | 0.002591774 | 0.001721663 | 0.002013176 |
| Furan, 2-pentyl- | Organoheterocyclic compounds | 0.006 |  | Green Beans, Vegetable | 12.33997038 | 13.43954763 | 3.46416472 | 5.873034169 |
| 2,3-Butanedione | Ketones | 0.002 | 2900 | pleasant, buttery | 46.00135255 | 27.4845924 | 1.408795905 | 16.88227882 |
| 1-Octen-3-one | Ketones | 0.005 |  | Mushroom-Like | 1.967498002 | 1.588142487 | 0.433151271 | 0.642035528 |
| Thiophene, 2-pentyl- | Organoheterocyclic compounds | 287 |  | Fruity, Sweet | 0 | 3.74115E-06 | 0 | 0 |
| Pentanoic acid, ethyl ester | Esters | 26.78 |  | Fruity, Apple-Flavor | 0.000460648 | 0.000162108 | 5.30107E-05 | 5.87865E-05 |
| 3-Heptanone, 5-methyl- | Ketones | 5900 |  | solvent, sharp | 1.74666E-07 | 1.88196E-07 | 2.70056E-08 | 1.20569E-08 |
| 2,6-Nonadienal, (E,Z)- | Aldehydes | 0.02 |  | Cucumber, Green | 0.168311389 | 0.082328065 | 0.031002911 | 0.035055304 |
| Terpinen-4-ol | Lipids and lipid-like molecules | 250 |  | Turpentine | 0 | 5.13538E-08 | 0 | 0 |
| Cyclohexanone, 2-methyl- | Ketones | 181 |  | acetone | 0 | 1.11567E-08 | 0 | 0 |
| Butanal, 3-methyl- | Aldehydes | 16.51 |  | Floral, Fruity | 0.000419155 | 0.000430349 | 0.000215312 | 0.000337937 |
| Pyrazine, 2,3-dimethyl- | Organoheterocyclic compounds | 10823.7 |  | Toasted Bread, Fried Corn, Roasted Buns, Roasted Peanuts | 0 | 0 | 0 | 1.32232E-09 |
| Phenylethyl Alcohol |  | 2600 |  | Floral | 1.95775E-07 | 2.35601E-07 | 7.61449E-09 | 1.03469E-08 |
| Thiophene, 3-methyl- | Organoheterocyclic compounds | 360 |  | Plastic, Sulfurous | 3.21521E-07 | 2.70216E-07 | 2.04749E-07 | 1.03744E-07 |
| Aniline | Benzenoids | 12 | 10000 | pungent, oily,empyreumatic | 1.45581E-05 | 5.5598E-06 | 1.89926E-06 | 4.81327E-06 |
| Methane, isocyanato- | Heterocyclic_Compounds | 2140 |  |  | 6.31291E-06 | 7.25351E-06 | 6.03554E-06 | 3.73091E-06 |
| Disulfide, dimethyl | Organosulfur compounds | 0.29 | 1450 | garlic, putrid,asparagus | 0.000108677 | 5.1924E-05 | 6.39001E-05 | 6.21792E-05 |
| Hexadecanoic acid, ethyl ester | Esters | 2 |  | Wax | 0.01577957 | 0.011451017 | 0.011399207 | 0.010567493 |
| 2-Nonanol | Alcohols | 50 |  | Rose | 0 | 6.24054E-07 | 0 | 0 |
| Ethanol | Alcohols | 24900 |  | Disolvent, Ethanol | 2.06017E-05 | 5.02474E-05 | 6.00313E-06 | 1.83157E-05 |
| 1,2-Propanediol, dinitrate | Organic oxygen compounds | 236 |  | disagreeable | 2.59794E-05 | 0 | 1.59992E-07 | 3.75135E-06 |
| Hexanal | Aldehydes | 20 |  | Green Grass, Fruity | 0.004448748 | 0.001924252 | 5.05209E-05 | 0.000249261 |
| Isopropyl Alcohol | Alcohols | 1000 | 2197000 | sharp, rubbingalcohol | 0 | 0 | 0 | 0.000125094 |
| Acetone | Ketones | 400 | 11745000 | sweet, fruity,etherous | 0.00040244 | 9.74357E-05 | 0.000272728 | 9.95239E-05 |
| 2-Hexenal, (E)- | Aldehydes | 190 |  | Apple-Like,Planty Green,Stinkbug | 6.68808E-05 | 6.9675E-05 | 7.45885E-06 | 8.91414E-06 |
| Acetic acid, mercapto- |  | 0.21 |  | unpleasant | 0 | 7.06817E-05 | 0 | 5.98496E-05 |
| 2H-Pyran-2-one, tetrahydro-6-propyl- | Organoheterocyclic compounds | 400 |  | Coconut | 8.3301E-08 | 0 | 0 | 0 |
| 1-Pentanol | Alcohols | 5.5 | 305000 |  | 0.012972568 | 0.010759349 | 0.003216958 | 0.004310493 |
| Benzene | Benzenoids | 470 | 313000 | aromatic, sweet,solvent,empyreumatic | 3.12772E-05 | 1.88389E-06 | 4.21062E-07 | 1.84426E-07 |
| 2-Propanamine |  | 25 | 700 | ammoniacal, amine | 0 | 0 | 0 | 0.000120988 |
| 2-Propanol, 2-methyl- | Alcohols | 3300 | 957000 | sweet alcohol | 0 | 3.26842E-07 | 8.19318E-08 | 4.5778E-07 |
| Boron trifluoride |  | 1500 |  | pungent | 7.91699E-07 | 3.19884E-07 | 1.33833E-07 | 7.43089E-08 |
| Linalool | Lipids and lipid-like molecules | 25 |  | Floral,Green | 1.45315E-07 | 0 | 0 | 0 |
| Propanal, 2-methyl- | Aldehydes | 0.34 | 139 | pungent | 0.000167474 | 0 | 0 | 0 |
| 2-Butanone | Ketones | 70 | 330900 | sweet, sharp | 0.000695617 | 0.00052553 | 0.000265016 | 0.000232357 |
| Ethane, 1,1,2,2-tetrachloro- | Organohalogen compounds | 233 | 7300 | solvent | 0 | 0 | 0 | 2.20905E-08 |
| 2-Propenoic acid, 2-methyl- | Carboxylic_Acids | 540 | 2840 | pungent | 9.86784E-08 | 0 | 0 | 0 |
| 2-Nonanone | Ketones | 15 |  | Fruity, Floral, Fatty | 5.57243E-05 | 7.92193E-05 | 2.37876E-05 | 3.56039E-05 |
| 1-Decene | Hydrocarbons | 6450 |  | pleasant | 1.51187E-07 | 8.14451E-08 | 3.67887E-08 | 2.64106E-08 |
| Benzothiazole | Organoheterocyclic compounds | 3871 |  | Gasoline,Leather, Roasted,Smoky And Rubber | 7.32736E-08 | 1.10134E-07 | 6.64379E-08 | 4.56016E-08 |
| Benzene, 1,2-dichloro- | Benzenoids | 20 | 50000 | camphor | 4.47514E-06 | 2.27712E-06 | 5.0663E-07 | 8.93613E-07 |
| Butanal, 2-methyl- | Aldehydes | 0.001 |  | Cocoa, Almond | 0.084465712 | 0 | 0.204224297 | 0.124629698 |
| Propanoic acid, 2-methyl-, ethyl ester | Esters | 4.5 |  | rubber, alcoholic, ethereal, fusel, strawberry, rummy, sweet | 0.000404162 | 2.44283E-05 | 6.38424E-06 | 5.81773E-05 |
| Propanoic acid, 2-hydroxy-, ethyl ester | Esters | 128000 |  | Fruity, Slightly Fattyflavor | 4.08944E-07 | 5.78002E-08 | 2.38651E-07 | 1.7586E-07 |
| 2-Thiophenecarboxaldehyde | Aldehydes | 8878 |  | Almond, Cherry | 0 | 0 | 1.9017E-09 | 1.38168E-09 |
| a-Terpineol | Lipids and lipid-like molecules | 1 |  | Piney, Iris, Teil | 0.000624788 | 0.000611029 | 9.6366E-05 | 0.000571807 |
| gamma-Terpinene | Hydrocarbons | 3260 |  | Oily, Smoky | 0 | 2.08038E-09 | 0 | 1.59498E-09 |
